# Site-comprehensive phosphorylation dismantles a disordered interaction hub to drive Cdc25C activation

**DOI:** 10.1016/j.isci.2026.118169

**Published:** 2026-11-05

**Authors:** Tan Tan, Chuanfen Wu, Ruoning Wang, Wei Zhang, Sue-Hwa Lin, Jian Kuang

**Affiliations:** 1 Department of Experimental Therapeutics, University of Texas MD Anderson Cancer Center, Houston, TX 77030, USA; 2 Department of Biochemistry and Molecular Biology, University of South China, Hengyang, Hunan 421001, China; 3 Key Laboratory of Cell Proliferation and Regulation Biology of the Ministry of Education, College of Life Sciences, Beijing Normal University, Beijing 100875, China; 4 Department of Translational Molecular Pathology, University of Texas MD Anderson Cancer Center, Houston, TX 77030, USA

**Keywords:** the M-phase supershift, biphasic regulation of Cdc25C, site-comprehensive phosphorylation, intrinsically disordered regulatory region, interaction network, *Xenopus* oocyte maturation, mitotic kinases

## Abstract

The Cdk1 activator Cdc25C contains a long intrinsically disordered regulatory region (IDR) that mediates the IDR-catalytic domain interaction, substrate recruitment via interaction with cyclin B, self-association, and heterotypic interactions with numerous partners. While site-comprehensive phosphorylation across the IDR dramatically activates Cdc25C at M-phase onset, prior site-specific phosphorylation produces only modest activation. Here, we show that the IDR functions as a phosphorylation-responsive interaction hub whose interaction state determines whether Cdc25C remains restrained or transitions to an active state. Before M-phase entry, site-specific phosphorylation selectively rewires the IDR-mediated interaction network, promoting cyclin B recruitment to modestly activate Cdc25C. At M-phase entry, site-comprehensive phosphorylation globally dismantles all four classes of IDR-mediated interactions to enable robust Cdc25C activation. These findings illustrate how site-comprehensive phosphorylation of long IDRs can function as a decisive biochemical switch and provide a mechanistic framework for understanding the biphasic regulation of Cdc25C activity during M-phase induction.

## Highlights

• The Cdc25C IDR functions as a phosphorylation-responsive interaction hub
• Engagement of numerous partners by the IDR restrains Cdc25C activity in G2 phase
• Site-specific phosphorylation modestly activates Cdc25C via cyclin B recruitment
• Site-comprehensive phosphorylation dismantles the hub for robust Cdc25C activation

## Graphical abstract

**Figure:**
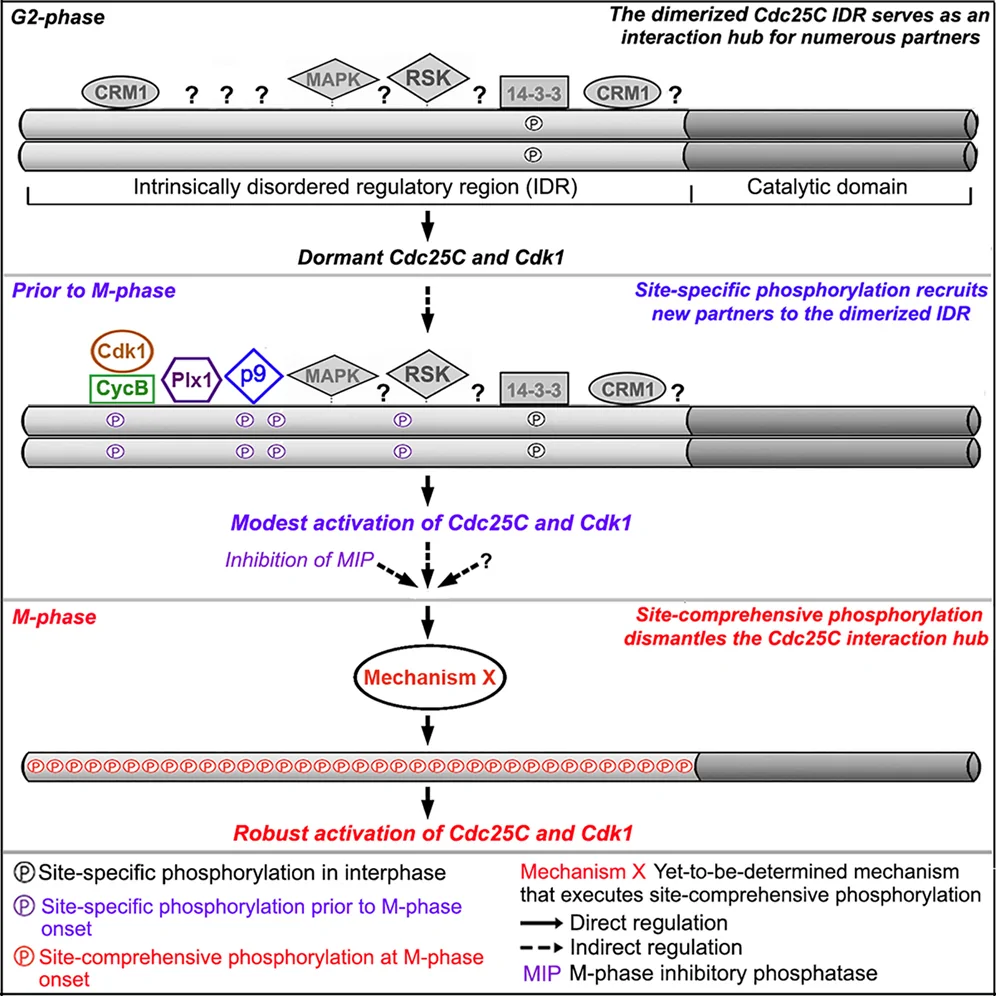

## Teaser

Biochemistry; Molecular biology; Cell biology

## Introduction

Entry into mitosis and meiosis (M-phase) is accompanied by extensive protein phosphorylation.^1^^,^^2^ A hallmark of this process is the pronounced gel mobility shift displayed by many phosphoproteins,^3^^,^^4^ which we collectively refer to as the M-phase supershift.^5^ The supershifts of key mitotic regulators have been attributed to multisite phosphorylation at S/TP motifs by the trimeric Cdk1/CycB/Cks complex,^6^^,^^7^^,^^8^ although definitive experimental evidence has remained lacking.

To define the molecular basis of the M-phase supershift, we previously reconstituted this phenomenon for several key mitotic regulators, including *Xenopus* Cdc25C, greatwall (Gwl), APC3, Myt1, and Wee1A, by phosphorylating recombinant proteins with M-phase-stabilized *Xenopus* egg extracts (MEE). These experiments revealed that their supershifts arise from heterogeneous phosphorylation of most S/T residues across specific long intrinsically disordered regulatory regions (IDRs) that are enriched in both S/T residues and S/TP motifs. We designated these regions as supershift domains and the accompanying widespread, site-nonspecific phosphorylation as site-comprehensive or comprehensive phosphorylation. Notably, this unusual phosphorylation type does not require any of the four major mitotic kinases in MEE, Cdk1, Plx1, MAPK, or RSK2, revealing a mechanistically distinct mode of mitotic phosphorylation.

Current models of mitotic regulation primarily emphasize site-specific phosphorylation by major mitotic kinases. Our discovery of site-comprehensive phosphorylation, however, introduces a second and mechanistically distinct layer of regulation. To compare the roles of these two phosphorylation modes, we recently analyzed their respective effects on the gel mobility shift and phosphatase activation of *Xenopus* Cdc25C, both *in vitro* and in oocytes.^9^ We found that site-specific phosphorylation by Cdk1 and other major mitotic kinases produces only minor gel shifts and modest activation shortly before M-phase entry. In contrast, site-comprehensive phosphorylation triggers the characteristic M-phase supershift and robust activation of Cdc25C at M-phase onset. Together, these findings establish a two-stage framework for Cdc25C phosphoregulation during M-phase induction.

The biphasic regulation of Cdc25C raises a fundamental mechanistic question: why do the two types of phosphorylation differentially affect Cdc25C activity? Canonically, site-specific phosphorylation often activates enzymes by inducing defined conformational changes that enhance substrate recognition or catalytic efficiency.^10^ However, the activating phosphorylation of Cdc25C occurs within its long IDR, which exists as a dynamic ensemble of conformations.^11^ Although Cdk1, Plx1, MAPK, and RSK2 each modestly activate G2-state Cdc25C, they target distinct sites distributed across different regions of the IDR.^9^ In contrast, robust activation of Cdc25C at M-phase onset is associated with heterogeneous and simultaneous phosphorylation of most S/T residues throughout the IDR.^9^ These observations suggest that Cdc25C activation may arise not from stabilization of a particular folded conformation but from changes in other properties of its IDR.

Long IDRs are enriched in molecular recognition elements that mediate interactions with proteins, RNA, and DNA.^12^^,^^13^^,^^14^ Consistent with the interaction-rich nature of long IDRs, bioinformatic analyses of the *Xenopus* Cdc25C IDR identified numerous predicted or experimentally defined protein-binding elements, including 40 short linear motifs (Figure S1) and 10 molecular recognition feature (MoRF) regions (Figure S2). Consistent with these predictions, *Xenopus* Cdc25C interacts with numerous regulatory proteins, including Raf1,^15^ 14-3-3,^16^ CRM1,^17^^,^^18^ PP2A,^19^ and MAPK^20^ (Figure 1A). Together, these observations suggest that its IDR serves as a platform for extensive protein interactions whose phosphorylation-induced remodeling may contribute to Cdc25C activation.

**Figure 1:**
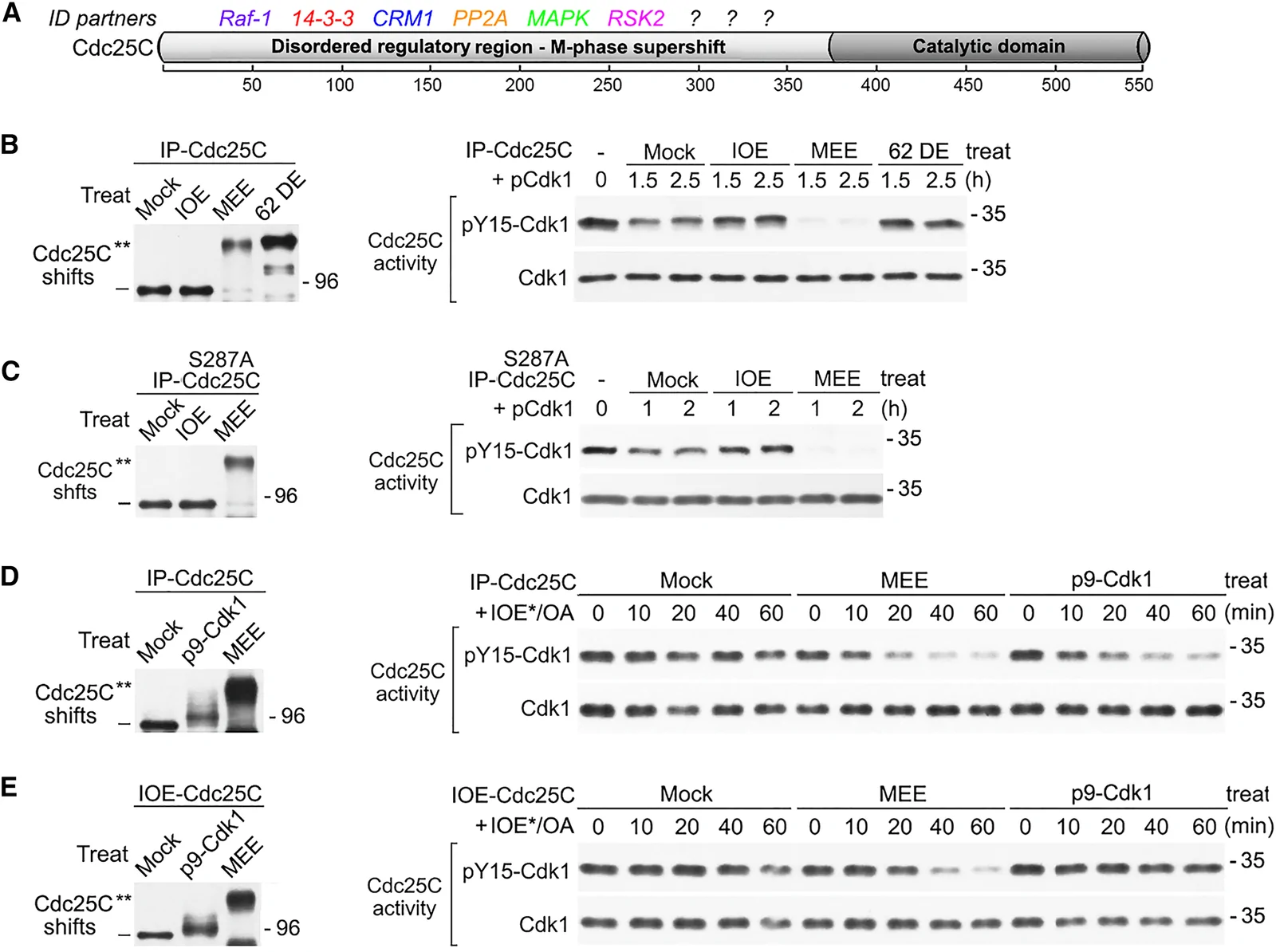
G2-state Cdc25C differs from original-state Cdc25C in basal activity and phosphoactivation (A) Schematic diagram of *Xenopus* Cdc25C interactions with binding partners. (B) Myc-Cdc25C immunoprecipitated from its TNT product (IP-Cdc25C) was mock-treated with EB or incubated with IOE or MEE for 1 h. The washed immunocomplexes were analyzed for Cdc25C mobility shifts by anti-Myc immunoblotting (left) and for Cdc25C activity by measuring dephosphorylation of partially purified phospho-Cdk1 (pCdk1) for the indicated times (right). In parallel, TNT-produced myc-Cdc25C 62DE was immunoprecipitated and analyzed. (C) IP-Cdc25C S287A was analyzed as described for IP-Cdc25C in (B). (D) IP-Cdc25C was mock-treated with EB or incubated with p9-Cdk1 or MEE for 1 h. Washed immunocomplexes were examined for Cdc25C mobility shifts by anti-Myc immunoblotting (left) and for Cdc25C activity by measuring dephosphorylation of endogenous Cdk1 in 1:10-diluted IOE⁎ supplemented with okadaic acid (IOE⁎/OA) for the indicated times (right). Similar results were obtained in four additional independent experiments with moderate variations in phosphorylation conditions. (E) IOE-Cdc25C (lower images) was mock-treated with EB or incubated with p9-Cdk1 or MEE for 1 h and analyzed as in (D). The results shown are representative of five independent experiments.

In this study, we performed a comprehensive analysis of the interaction network mediated by the Cdc25C IDR and investigated how the two phosphorylation modes influence this network *in vitro* and in *Xenopus* oocytes. We identified four classes of IDR-mediated interactions, including intramolecular interaction with the catalytic domain, substrate recruitment through cyclin B binding, homotypic self-association, and heterotypic interactions with numerous binding partners. Whereas site-specific phosphorylation uniquely promotes interactions of G2-state Cdc25C with cyclin B, Plx1, and Cks proteins, site-comprehensive phosphorylation disrupts all four classes of IDR-mediated interactions. Together, these findings provide a mechanistic basis for understanding the biphasic regulation of Cdc25C activity during M-phase induction.

## Results

### G2-state Cdc25C differs from original-state Cdc25C in basal activity and responsiveness to activating phosphorylation

Previous studies examining the phosphoregulation of Cdc25C activity *in vitro* have typically used purified recombinant Cdc25C in its uncomplexed form.^20^^,^^21^^,^^22^^,^^23^^,^^24^ Because *Xenopus* Cdc25C associates with multiple binding partners during G2 phase (Figure 1A), its basal activity may differ from that of the newly synthesized, unbound Cdc25C, hereafter referred to as original-state Cdc25C. To test this possibility, we prepared Cdc25C immunocomplexes representing the original, G2, and M states and compared their Cdk1 phosphatase activities (Figure S3A).

Original-state Cdc25C (IP-Cdc25C) was prepared by immunoprecipitating myc-Cdc25C directly from the transcription-translation (TNT) reaction, whereas incubation of IP-Cdc25C with immature oocyte extract (IOE) or MEE generated G2-state (IOE-Cdc25C) and M-state (MEE-Cdc25C) Cdc25C, respectively. Stage V and VI immature oocytes are naturally arrested in G2 phase, and IOE was prepared without strong phosphatase inhibition. In contrast, MEE was prepared with strong phosphatase inhibitors such as okadaic acid (OA) and NaF, as well as ATP-γ-S to generate phosphatase-resistant thiophosphorylation, resulting in a phosphorylation-dephosphorylation balance different from that in M-phase-arrested *Xenopus* eggs. Nevertheless, our previous studies showed that MEE faithfully recapitulates the major phosphorylation events that produce the M-phase supershift of Cdc25C,^5^ making it a suitable experimental system for reconstituting M-phase-specific site-comprehensive phosphorylation of Cdc25C. Phosphatase activity was assessed using partially purified Y15-phosphorylated Cdk1 (pCdk1; Figure S3B). For comparison, we also examined the previously described 62DE mutant, in which all 62 Ser/Thr residues within the IDR were replaced with Asp/Glu. Although each Asp/Glu substitution introduces only half the negative charge of a phosphate group,^25^ this across-the-board phosphomimetic mutation produces a pronounced gel mobility shift comparable to that induced by MEE.^5^^,^^9^

As shown in Figure 1B, mock-treated IP-Cdc25C exhibited detectable Cdk1-dephosphorylating activity, as indicated by reduced pY15-Cdk1 at both time points. IOE treatment modestly decreased this basal activity, whereas MEE treatment dramatically enhanced the activity. These results recapitulate the robust activation of Cdc25C during M-phase entry and suggest that post-translational modifications and/or partner interactions associated with the G2 state contribute to the reduced basal activity of Cdc25C. Interestingly, the 62DE mutation decreased the basal activity of original-state Cdc25C to a level comparable to that of IOE-treated Cdc25C, consistent with our previous results.^9^ These findings indicate that the 62DE mutation not only fails to recapitulate the activating effects of authentic site-comprehensive phosphorylation but also introduces inhibitory features that suppress Cdc25C activity. Thus, the 62DE mutant cannot be used to infer the mechanism of Cdc25C activation.

To determine whether S287 phosphorylation, which promotes 14-3-3 binding and modestly reduces Cdc25C activity,^16^ accounts for the reduced basal activity of G2-state Cdc25C, we analyzed the S287A mutant. As shown in Figure 1C, IOE treatment still modestly decreased the activity of S287A-Cdc25C relative to the original state, indicating that S287 phosphorylation-dependent 14-3-3 recruitment alone cannot explain the lower basal activity of G2-state Cdc25C.

We next asked whether G2-state Cdc25C also responds differently to activating phosphorylation than original-state Cdc25C. IP-Cdc25C or IOE-Cdc25C was phosphorylated with either MEE or an affinity-purified Cdk1/cyclin B complex saturated with the *Xenopus* CKS protein p9 (p9-Cdk1), which displayed H1 kinase activity comparable to that of MEE.^5^ The treated myc-Cdc25C immunocomplexes were then assayed for endogenous Cdk1 dephosphorylation in 1:10-diluted high-quality IOE (IOE∗) supplemented with 1 μM OA (IOE∗/OA) without endogenous Cdc25C activation.^5^ While phosphorylation with p9-Cdk1 induced a clear gel mobility shift in both IP-Cdc25C (Figure 1D) and IOE-Cdc25C (Figure 1E), IP-Cdc25C exhibited a marked increase in activity, whereas IOE-Cdc25C showed little or no increase, consistent with our previous results.^9^ In contrast, MEE-induced phosphorylation, which produced substantially larger mobility shifts, strongly activated both IP-Cdc25C and IOE-Cdc25C. Although the physiological relevance of p9-Cdk1 remains unclear, these findings indicate that G2-state Cdc25C has a higher phosphorylation threshold for activation than original-state Cdc25C.

### Site-comprehensive phosphorylation activates original-state Cdc25C more efficiently than site-specific phosphorylation

Although original-state Cdc25C likely exists only transiently under physiological conditions, understanding how mitotic phosphorylation regulates this state may reveal fundamental principles governing Cdc25C activation. To address this question, we first compared the effects of site-specific and site-comprehensive phosphorylation on the activity of original-state Cdc25C.

Active Cdk1/CycB complex, Plx1, and a MAPK/RSK2 mixture (MAPK/RSK), the major mitotic kinases in MEE that phosphorylate distinct sites within the Cdc25C IDR (Figure S4A),^5^ were generated by *in vitro* TNT at levels comparable to those in MEE (Figures S4B and S4C). When incubated with immobilized GST-Cdc25C_9-374_ or GST-Cdc25C_375-550ˆ_, Cdk1/CycB and Plx1 preferentially associated with the IDR fragment, whereas MEK, MAPK, and RSK2 exhibited little or no interactions with either fragment (Figure S4D). These results indicate that the long IDR of original-state Cdc25C preferentially interacts with Cdk1/CycB and Plx1.

The TNT-derived kinase preparations were then used to phosphorylate IP-Cdc25C before phosphatase assays. As shown in Figure 2A, phosphorylation of IP-Cdc25C with MEE dramatically enhanced its Cdk1-dephosphorylating activity, as evidenced by near-complete dephosphorylation of pY15-Cdk1 at both the 1-h and 2-h time points. In contrast, phosphorylation with individual TNT-derived kinase preparations produced only modest increases in activity that were detectable primarily at the 2-h time point. Similar results were obtained when both the TNT-derived kinase preparations and MEE were diluted 4-fold (Figure 2B). Combining TNT-derived Cdk1/CycB, MAPK/RSK2 mix, and Plx1 preparations (4K mix) also elicited only modest activation, with minimal additive effects, whereas 1:4-diluted MEE continued to induce robust activation (Figure 2C). Together, these results indicate that MEE-mediated site-comprehensive phosphorylation activates original-state Cdc25C much more efficiently than site-specific phosphorylation by the four major mitotic kinases.

**Figure 2:**
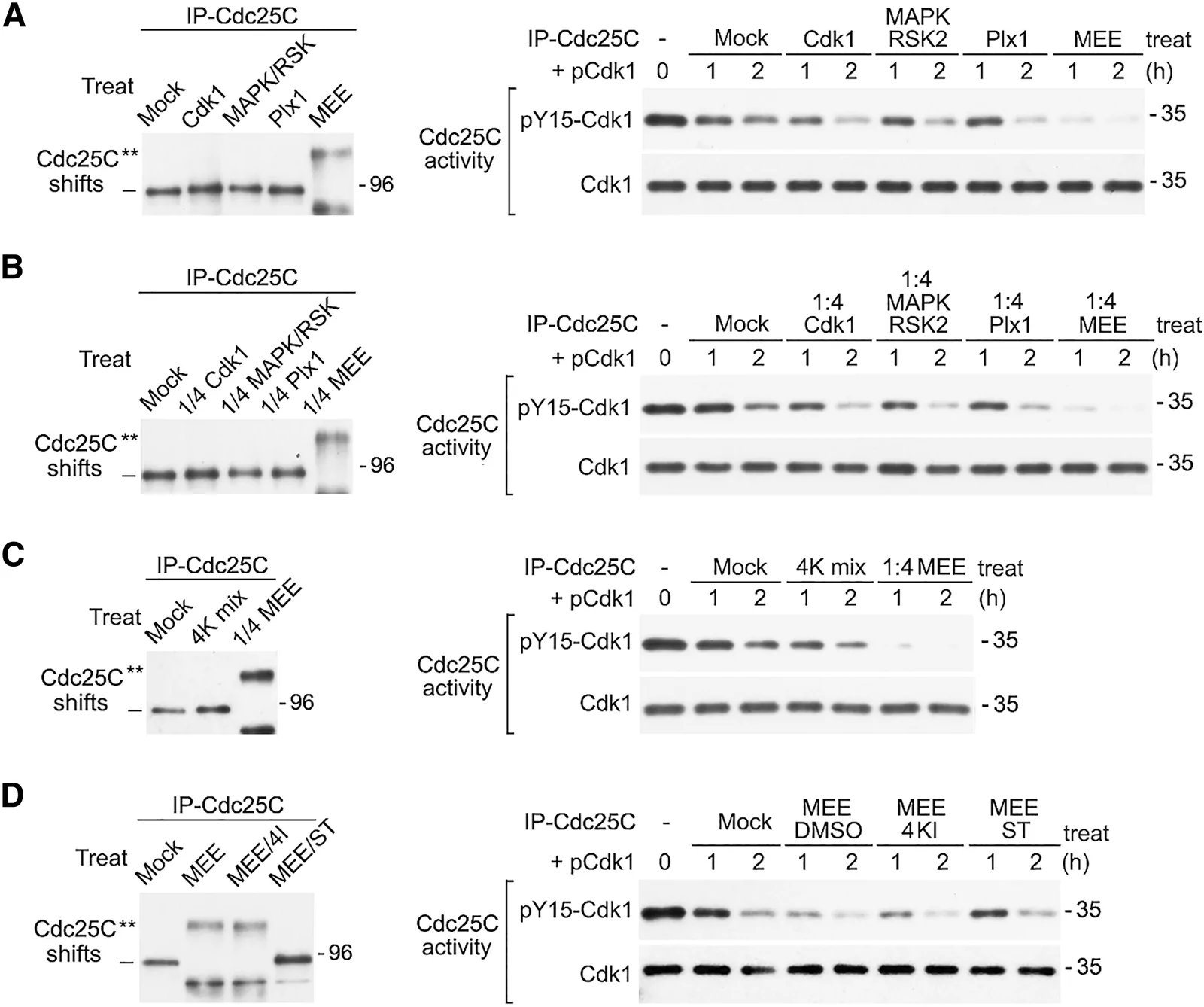
Site-specific and site-comprehensive phosphorylation regulate original-state Cdc25C activity differently (A) Myc-Cdc25C immunoprecipitated from its TNT product (IP-Cdc25C) was mock-treated with EB or incubated with individual TNT-produced kinases or MEE for 2 h. Washed immunocomplexes were analyzed for Cdc25C mobility shifts by anti-Myc immunoblotting (left) and for Cdc25C activity by measuring dephosphorylation of pCdk1 for the indicated times (right). The results shown are representative of two independent experiments. (B) IP-Cdc25C was mock-treated with EB or incubated with 1:4-diluted TNT kinase samples or 1:4-diluted MEE for 2 h, and immunocomplexes were analyzed as in (A). (C) IP-Cdc25C was mock-treated with EB or incubated with either a mixture of four TNT kinases (4K mix) or 1:4-diluted MEE for 2 h, and immunocomplexes were analyzed as in (A). (D) IP-Cdc25C was mock-treated with EB or incubated with MEE in the presence or absence of either the four specific kinase inhibitors (4KI) or the pan-kinase inhibitor staurosporine (ST) for 2 h. Washed immunocomplexes were analyzed as in (A).

To determine whether robust activation of original-state Cdc25C by MEE-mediated site-comprehensive phosphorylation requires any of the four major mitotic kinases, we phosphorylated IP-Cdc25C with MEE in the presence or absence of specific inhibitors targeting these kinases (4KI) or the pan-kinase inhibitor staurosporine (ST), followed by phosphatase assays. Whereas ST abolished both the MEE-induced mobility shift and robust activation of IP-Cdc25C, 4KI had little effect (Figure 2D), consistent with our previous results using IOE-Cdc25C.^9^ These results indicate that robust activation of original-state Cdc25C by MEE-mediated site-comprehensive phosphorylation does not require the activities of the four major mitotic kinases.

### Regulation of the IDR-catalytic domain interaction in original-state Cdc25C

To investigate whether the IDR-catalytic domain interaction contributes to the reduced basal activity of G2-state Cdc25C and its differential regulation by the two mitotic phosphorylation modes, we first characterized the interaction between the long IDR and the catalytic domain of original-state Cdc25C. Although none of the prior studies examined this interaction, full-length human Cdc25C and its isolated catalytic domain exhibit similar basal phosphatase activities,^26^ suggesting that the long IDR does not measurably suppress the basal activity of the catalytic domain.

Myc-tagged Cdc25C_9-550_, Cdc25C_9-375_, or Cdc25C_375-550_ was produced by TNT (Figure 3A) and assayed for binding to GST-tagged catalytically inactive Cdc25C_375-550_ (C457S, denoted by ˆ) using GST pulldown assays (Figure 3B). GST-Cdc25C_375-550ˆ_ readily pulled down both myc-Cdc25C_9-550_ and myc-Cdc25C_9-375_, but not myc-Cdc25C_375-550_, supporting the existence of an IDR-catalytic domain interaction. To map the interaction regions within the long IDR, Cdc25C_9-375_ was divided into three consecutive fragments: Cdc25C_9-129_, Cdc25C_126-213_, and Cdc25C_212-375_ (Figure 3A), and each fragment was tested for binding to GST-Cdc25C_375-550ˆ_ in parallel with Cdc25C_9-375_ via pulldown assays. All three fragments exhibited substantially weaker binding to GST-Cdc25C_375-550ˆ_ than did Cdc25C_9-375_, and a representative experiment is shown in Figure 3C. Although the relative pulldown intensities of each fragment varied among the three independent experiments, these results indicate that the long IDR interacts with the catalytic domain through multivalent contacts.

**Figure 3:**
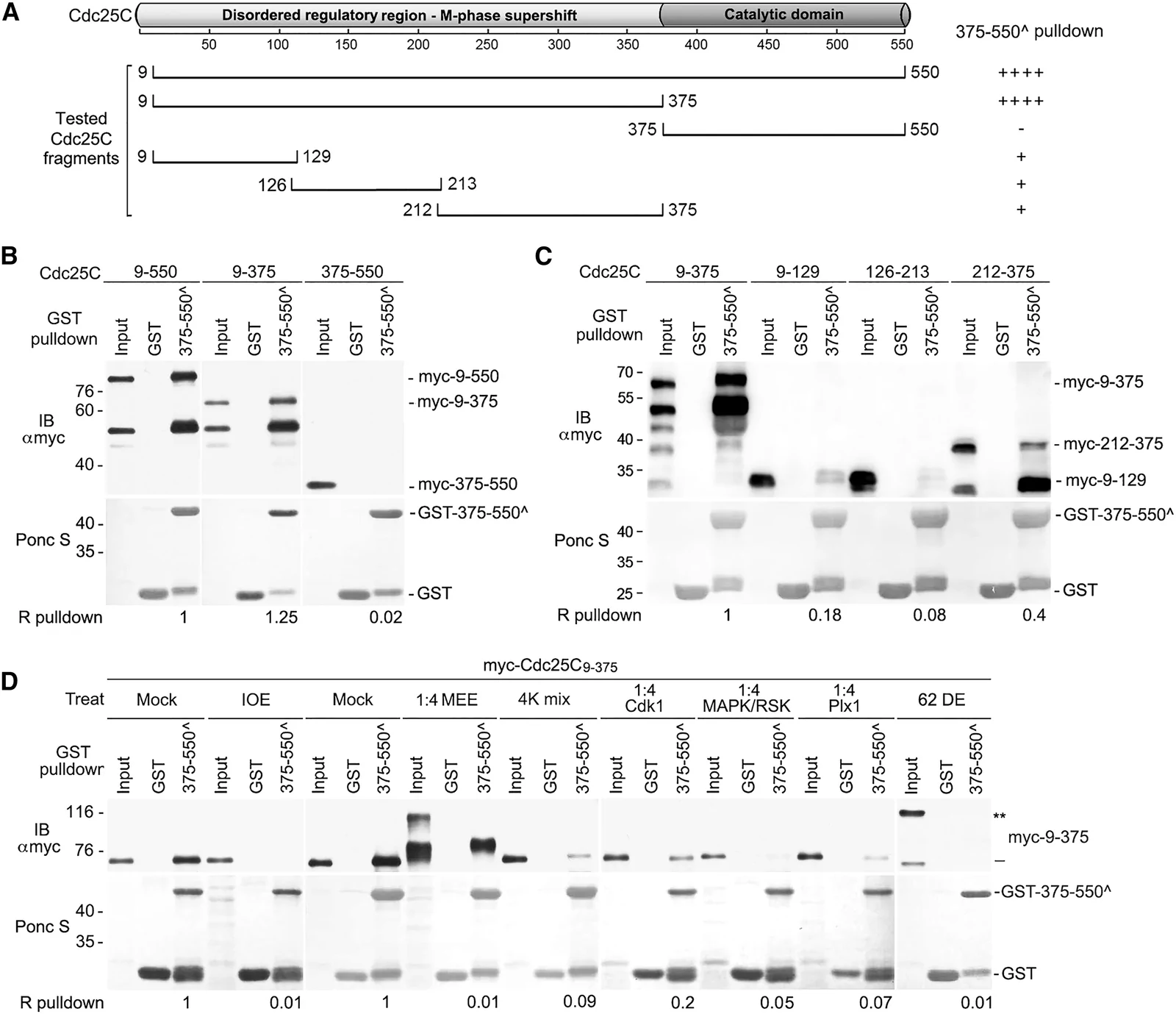
Both G2-state conversion and mitotic phosphorylation inhibit the regulatory-catalytic domain interaction of Cdc25C (A) Schematic illustration of Cdc25C structure and summary of results from testing various myc-tagged Cdc25C proteins for interaction with GST-Cdc25C_375-550ˆ_. (B) Myc-tagged Cdc25C_9-550_, Cdc25C_9-375_, and Cdc25C_375-550_ were each adsorbed with GST or GST-Cdc25C_375-550ˆ_ beads for 90 min. Input proteins (1/20) and bead eluates were analyzed by anti-Myc immunoblotting after Ponceau S (Ponc S) staining. Relative pulldown values (R pulldown) were calculated by normalizing the pulldown signal to the corresponding input signal and expressed relative to the indicated control, which was set to 1. (C) Myc-tagged Cdc25C_9-375_ (9–375), Cdc25C_9-129_ (9–129), Cdc25C_126-213_ (126–213), and Cdc25C_212-375_ (212–375) were processed as in (B). The results shown are representative of three independent experiments. (D) Myc-Cdc25C_9-375_ was mock-treated with EB or incubated with IOE, MEE, or the indicated TNT kinase samples for 2 h. Treated myc-Cdc25C_9-375_ and untreated myc-Cdc25C_9-375_ 62DE were analyzed as in (B). Similar results were obtained in an additional experiment using undiluted MEE or individual TNT-produced kinases for phosphorylation.

Further characterization of the IDR-catalytic domain interaction showed that GST-Cdc25C_375-550ˆ_, but not GST-Cdc25C_205-550ˆ_, pulled down myc-Cdc25C_9-213_, indicating that the IDR segment in Cdc25C_205-550ˆ_ masks the adjacent catalytic domain through an intramolecular interaction (Figure S5A). In contrast to GST-Cdc25C_375-550ˆ_, GST-Cdc25C_205-375_ failed to pull down either myc-Cdc25C_9-213_ or myc-Cdc25C_9-129_ (Figure S5B), indicating that different regions in the IDR do not interact with each other. Unlike the Cdc25C IDR, supershift domains of Wee1A (Wee1A_1-199_) and Gwl (Gwl_179-745_), which share structural features with the Cdc25C supershift domain,^5^ exhibited minimal or no binding to GST-Cdc25C_375-550ˆ_ (Figure S5C), demonstrating that the catalytic domain of Cdc25C interacts specifically with its own IDR.

Having established that the long IDR of Cdc25C forms multivalent contacts with its own catalytic domain, we next examined how G2-state conversion and site-specific versus site-comprehensive phosphorylation affect this interaction (Figure 3D). Myc-Cdc25C_9-375_ was incubated with either IOE, MEE, or 1:4-diluted TNT-derived mitotic kinases, after which its binding to GST-Cdc25C_375-550ˆ_ was assessed by GST pulldown. In parallel, the phosphomimetic 62DE mutant was analyzed without prior phosphorylation. Both IOE and MEE treatments abolished binding, and these results were confirmed by a reciprocal pulldown assay in which immunoprecipitated myc-Cdc25C_9-375_ was used to pull down soluble GST-Cdc25C_375-550ˆ_ (Figures S6A and S6B). The 62DE mutation similarly abolished binding. Moreover, site-specific phosphorylation by 1:4-diluted Cdk1, Plx1, MAPK/RSK, and the 4K mix also markedly weakened this interaction, although not to the same extent as 1:4-diluted MEE. To examine the individual effects of MAPK and RSK2, we phosphorylated immunoprecipitated myc-Cdc25C_9-375_ with MAPK/RSK, MAPK alone, or MAPK/RSK in the presence of the MAPK inhibitor VX-11e (VX), which selectively blocked phosphorylation at the MAPK site (S163) (Figure S6C) and examined pulldown of soluble GST-Cdc25C_375-550ˆ_. All three phosphorylation conditions reduced pulldown of soluble GST-Cdc25C_375-550ˆ_ (Figure S6D), although to a lesser extent than MEE (Figure S6B), indicating that MAPK and RSK2 each independently weakened the IDR-catalytic domain interaction. Because G2-state conversion, site-specific phosphorylation, and site-comprehensive phosphorylation relieved the IDR-catalytic domain interaction to broadly similar extents despite their markedly different effects on Cdc25C activity, disruption of this interaction is unlikely to be a critical event underlying Cdc25C activation.

### Unique roles of site-comprehensive phosphorylation in inhibiting Cdc25C-cyclin B interaction

Previous studies demonstrated that cyclin B binding to Cdc25C is required for its modest activation preceding M-phase entry but becomes dispensable for its robust activation within M phase.^27^ Mechanistically, cyclin B binding has been proposed both to directly enhance Cdc25 phosphatase activity^28^ and to position the substrate Cdk1/CycB in proximity to Cdc25C.^29^ To determine whether cyclin B binding contributes to Cdc25C phosphoactivation, we next examined how G2-state conversion, site-specific phosphorylation, and site-comprehensive phosphorylation regulate the interaction between Cdc25C and cyclin B.

Analysis using the Eukaryotic Linear Motif (ELM) resource identified two RxL-type cyclin-binding motifs within the long IDR of *Xenopus* Cdc25C at residues 67–75 and 334–348 (or 337–348) (Figures 4A and S7A). In addition, a 51-residue peptide (MP51) from human Cdc25C, corresponding to residues 263–315 of *Xenopus* Cdc25C, has been shown to bind cyclin B independently of its phosphorylation-dependent interaction with 14-3-3 (Figures 4A and S7).^30^ Together, these observations suggest that the long IDR of Cdc25C contains multiple cyclin B-binding sites. To test this possibility, myc-tagged Cdc25C_9-550_, Cdc25C_9-375,_ and Cdc25C_375-550_ were produced by TNT (Figure 4A) and assayed for binding to GST-tagged *Xenopus* cyclin B2 (GST-CycB2) by pulldown (Figure 4B). GST-CycB2 pulled down myc-Cdc25C_9-550_ and myc-Cdc25C_9-375_, but not myc-Cdc25C_375-550_, indicating that cyclin B binding is mediated by the IDR. To map the binding regions within the IDR, myc-tagged Cdc25C_9-129_, Cdc25C_126-213_, and Cdc25C_212-375_ were tested in parallel with Cdc25C_9-375_ (Figure 4A). Although the relative binding of the individual fragments varied among experiments, all three fragments bound GST-CycB2 much more weakly than did Cdc25C_9-375_, as exemplified in Figure 4C. These results indicate that the long IDR engages cyclin B through multiple weak interactions.

**Figure 4:**
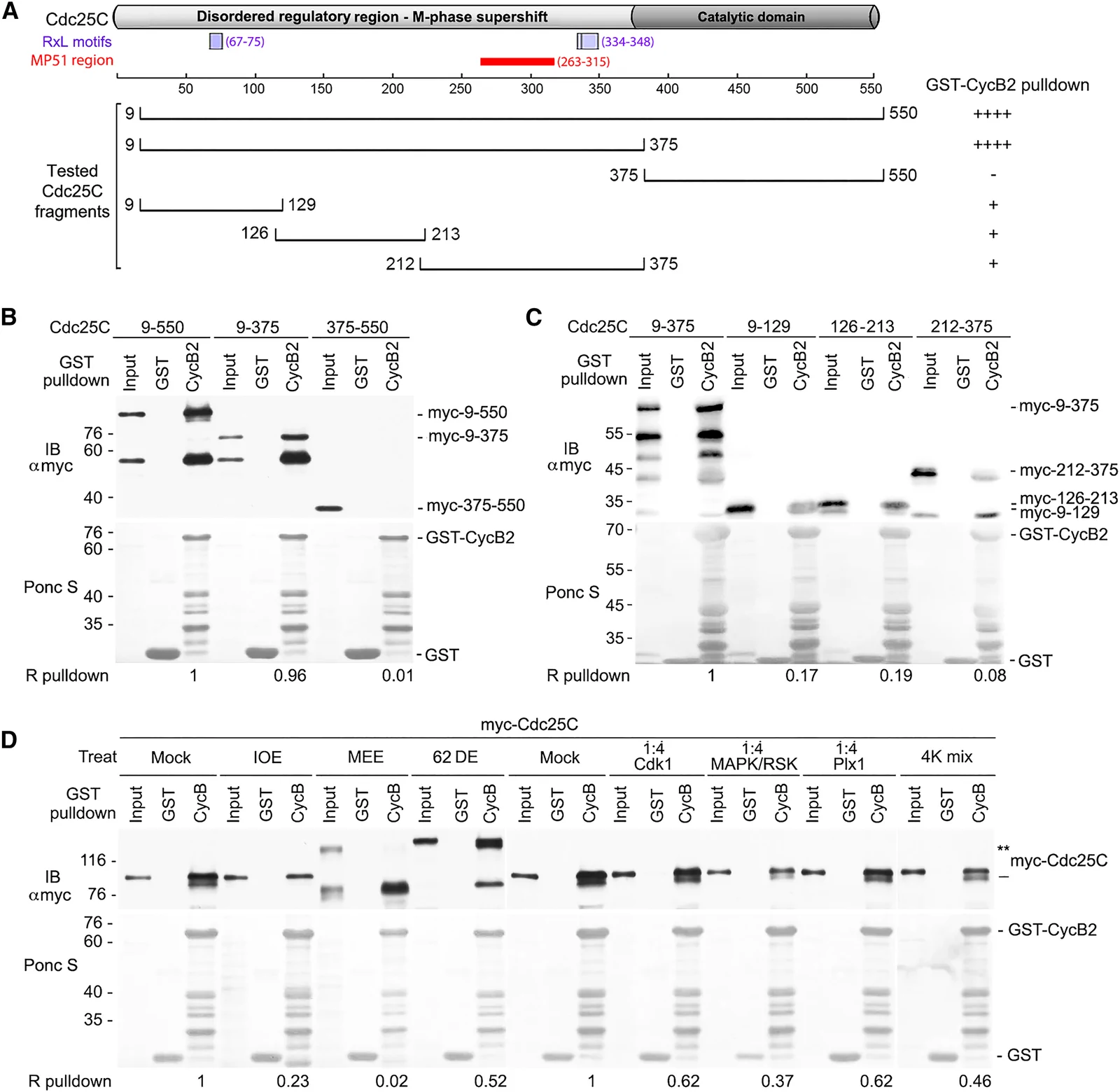
Cdc25C IDR interaction with cyclin B is inhibited by G2-state conversion and site-comprehensive phosphorylation (A) Schematic diagram of Cdc25C structure and summary of results from testing various Cdc25C proteins for interaction with GST-tagged *Xenopus* cyclin B2 (GST-CycB2). (B) Myc-tagged Cdc25C_9-550_ (9–550), Cdc25C_9-375_ (9–375), and Cdc25C_375-550_ (375–550) were each adsorbed with GST or GST-CycB2 beads for 90 min. Input proteins (1/20) and bead eluates were analyzed by anti-Myc immunoblotting after Ponceau S (Ponc S) staining. Relative pulldown values (R pulldown) were calculated by normalizing the pulldown signal to the corresponding input signal. Values are expressed relative to the indicated control, which was set to 1. (C) Myc-tagged Cdc25C_9-375_ (9–375), Cdc25C_9-129_ (9–129), Cdc25C_126-213_ (126–213), and Cdc25C_212-375_ (212–375) were processed as in (B). The results shown are representative of three independent experiments. (D) Myc-tagged Cdc25C_9-550_ (myc-Cdc25C) was mock-treated with EB or incubated with IOE, MEE, or the indicated TNT kinase samples for 2 h. Treated myc-Cdc25C and untreated myc-Cdc25C 62DE were analyzed as in (B).

Having established that the long IDR of Cdc25C forms multivalent contacts with cyclin B, we next examined how G2-state conversion, site-specific phosphorylation, and site-comprehensive phosphorylation affect this interaction. Myc-Cdc25C was incubated with either IOE, MEE, or 1:4-diluted TNT-produced individual or combined mitotic kinases, and its binding to GST-CycB2 was assessed by pulldown. In parallel, the 62DE mutant was analyzed without prior phosphorylation (Figure 4D). IOE treatment reduced cyclin B binding by >4-fold. A reciprocal pulldown assay, in which immunoprecipitated myc-Cdc25C was used to capture soluble GST-CycB2, showed an approximately 7-fold reduction in cyclin B binding in response to IOE treatment (Figure S7B). Since cyclin B binding has been shown to be required for the modest activation of Cdc25C preceding M-phase entry,^27^ dramatically reduced cyclin B binding may contribute to the reduced basal activity of G2-state Cdc25C.

Although both MEE treatment and the 62DE mutation produced a supershift, MEE treatment largely abolished cyclin B binding, whereas the 62DE mutation, which introduces fewer negative charges and no phosphate groups, reduced cyclin B binding by only ∼2-fold. These findings suggest that the IDR-cyclin B interaction is more robust than the IDR-catalytic domain interaction. In contrast to the abolishing effect of MEE treatment, site-specific phosphorylation by individual or combined mitotic kinases modestly reduced cyclin B binding by about 2-fold. Since their differential effects on cyclin B association correlate with their distinct effects on Cdc25C activation (Figure 2), disruption of the IDR-cyclin B interaction may contribute to robust activation of Cdc25C.

### Unique roles of site-comprehensive phosphorylation in disrupting self-association of original-state Cdc25C

Because gel filtration analysis of purified recombinant human Cdc25C suggested that the protein forms oligomers,^31^ we next asked whether the IDR of original-state *Xenopus* Cdc25C self-associates. To address this question, myc-Cdc25C_9-375_ and HA-Cdc25C_9-375_ were produced separately by TNT, and immunoprecipitated myc-Cdc25C_9-375_ was used to pull down soluble HA-Cdc25C_9-375_ under different wash conditions. As shown in Figure 5A, soluble HA-Cdc25C_9-375_ readily associated with immunoprecipitated myc-Cdc25C_9-375_, and the interaction persisted after stringent washes containing high salt (500 mM NaCl), high detergent (0.5% Triton X-100), or both. Similar results were obtained when HA- and myc-tagged Cdc25C_9-375_ were co-produced in the same TNT reaction before immunoprecipitation (Figure S8A). In contrast, co-produced HA- and myc-tagged MAPK failed to co-immunoprecipitate under identical wash conditions (Figure S8B). Together, these results demonstrate that the IDR of original-state Cdc25C possesses a strong capacity for self-association.

**Figure 5:**
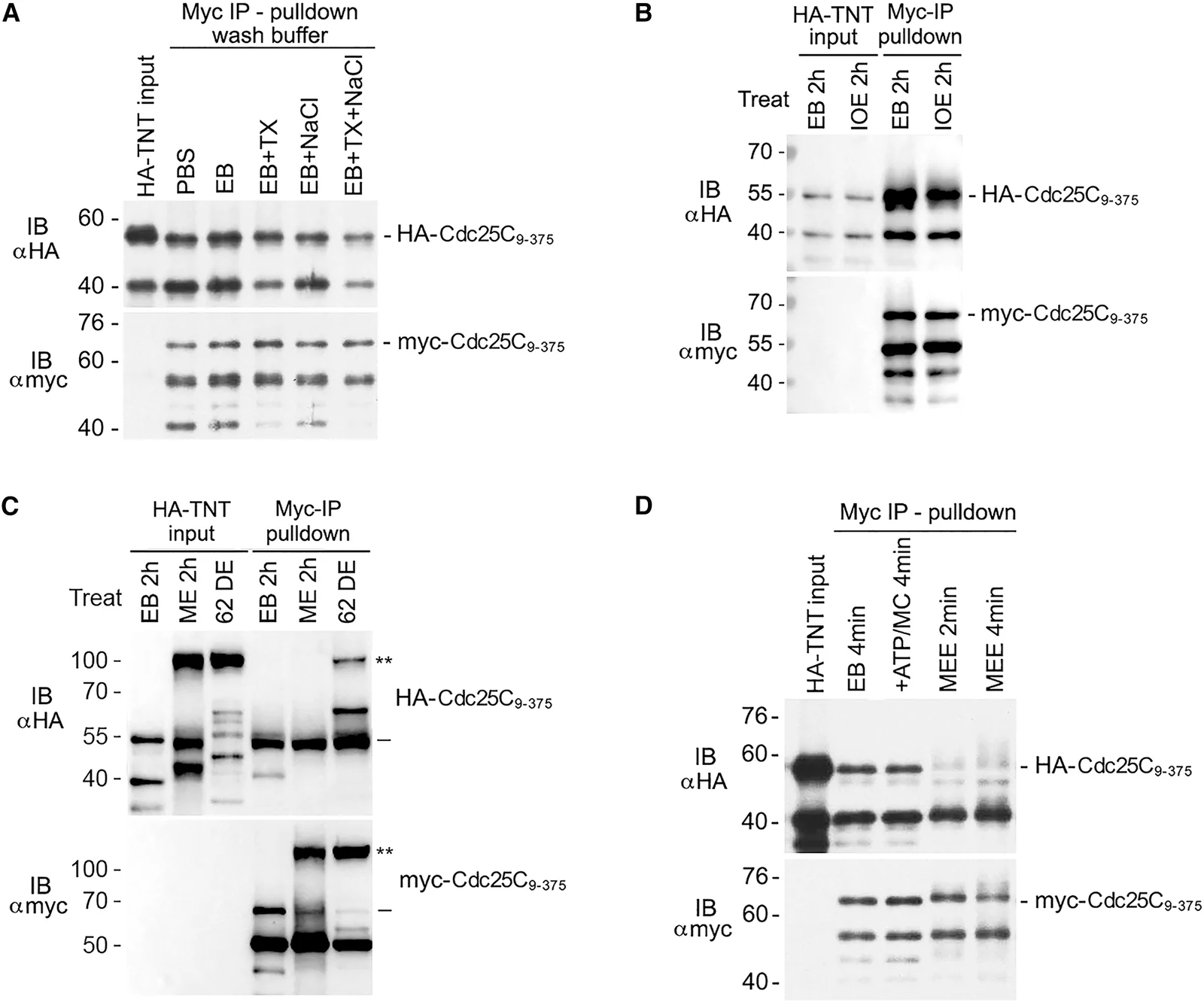
Site-comprehensive phosphorylation disrupts IDR-mediated self-association of Cdc25C (A) TNT-produced soluble HA-Cdc25C_9-375_ (HA-TNT input) was adsorbed with immunoprecipitated myc-Cdc25C_9-375_ for 1 h. Following two bead washes with the indicated buffers, HA-TNT input (1/10) and bead eluates (Myc-IP pulldown) were analyzed by immunoblotting with anti-Myc and anti-HA antibodies. TX, 0.5% Triton X-100; NaCl, 500 mM NaCl. (B) Soluble HA-Cdc25C_9-375_ and immunoprecipitated myc-Cdc25C_9-375_ were each mock-treated with EB or incubated with IOE for 2 h, and equivalently treated pairs were then incubated together for 1 h. Following two washes of beads with EB, bead eluates (Myc-IP pulldown) were analyzed in parallel with HA-TNT input (1/20) by immunoblotting with anti-Myc and anti-HA antibodies. The results shown are representative of two independent experiments. (C) Soluble HA-Cdc25C_9-375_ and immunoprecipitated myc-Cdc25C_9-375_ were each mock-treated with EB or incubated with mitotic extract (ME) derived from synchronized HEK293T cells for 2 h, whereas soluble HA-Cdc25C_9-375_ 62DE and immunoprecipitated myc-Cdc25C_9-375_ 62DE were untreated. Equivalently treated pairs were then incubated together for 1 h, and samples were processed as in (B). The results shown are representative of two independent experiments. (D) TNT-produced soluble HA-Cdc25C_9-375_ (HA-TNT input) was adsorbed with immunoprecipitated myc-Cdc25C_9-375_ for 1.5 h, and pelleted beads were incubated with two volumes of EB, EB/ATP/MC (microcystin), or MEE for 2 or 4 min. Following two bead washes with EB, HA-TNT input (1/10) and bead eluates (Myc-IP pulldown) were analyzed by immunoblotting with anti-Myc and anti-HA antibodies.

To determine whether G2-state conversion affects Cdc25C self-association, both soluble HA-Cdc25C_9-375_ and immunoprecipitated myc-Cdc25C_9-375_ were incubated with IOE for 2 h before the pulldown assays. As shown in Figure 5B, IOE treatment did not affect the interaction between HA- and myc-tagged Cdc25C_9-375_. Likewise, full-length catalytically inactive Cdc25Cˆ retained self-association after IOE treatment (Figure S8C). These results indicate that G2-state conversion does not disrupt Cdc25C self-association.

We next examined whether site-comprehensive phosphorylation disrupts Cdc25C self-association. To this end, both soluble HA-Cdc25C_9-375_ and immunoprecipitated myc-Cdc25C_9-375_ were incubated with mitotic extract (ME) derived from synchronized HEK293T cells for 2 h before the pulldown assay. In parallel, HA- and myc-tagged Cdc25C_9-375_ 62DE were analyzed under the same conditions. As shown in Figure 5C, ME-induced site-comprehensive phosphorylation abolished the interaction between HA- and myc-tagged Cdc25C_9-375_, whereas the 62DE mutation failed to do so. Similar results were obtained with full-length catalytically inactive Cdc25Cˆ (Figure S8D). Together, these results indicate that authentic site-comprehensive phosphorylation, but not the comprehensive phosphomimetic mutation, disrupts Cdc25C self-association.

To further determine whether disruption of Cdc25C self-association requires completion of site-comprehensive phosphorylation, coimmunoprecipitated HA-Cdc25C_9-375_ and myc-Cdc25C_9-375_ were briefly incubated with MEE for 2 or 4 min to allow partial progression of site-comprehensive phosphorylation. As shown in Figure 5D, partially phosphorylated HA-Cdc25C_9-375_ still bound partially phosphorylated myc-Cdc25C_9-375_, indicating that efficient disruption of self-association requires site-comprehensive phosphorylation.

### Cdc25C IDR does not intrinsically undergo liquid-liquid phase separation

Because the long Cdc25C IDR mediates self-association, we next asked whether Cdc25C undergoes liquid-liquid phase separation. *Xenopus* Cdc25C was fused to C-terminal GFP and His tags for fluorescence imaging and purification, respectively (Figure S9A). Purified Cdc25C-GFP-His was examined by fluorescence microscopy in the presence or absence of 12.5% polyethylene glycol (PEG) as a molecular crowding agent. As shown in Figure S9B, no condensate formation was detected at a protein concentration of ∼46 μM in extraction buffer (EB) or EB supplemented with 500 mM NaCl, whereas 12.5% PEG induced droplet formation. These results indicate that the Cdc25C IDR does not undergo liquid-liquid phase separation in the absence of macromolecular crowding and support a model in which site-comprehensive phosphorylation primarily regulates Cdc25C protein interactions rather than Cdc25C condensate formation.

### Unique roles of site-comprehensive phosphorylation in dissociating prebound partners from G2-state Cdc25C

Because partner occupancy of the long IDR in G2-state Cdc25C both suppresses basal activity and raises the threshold for phosphoactivation, we hypothesized that its robust activation requires dissociation from prebound partners. To test this idea, we determined how site-specific and site-comprehensive phosphorylation affect the interaction between Cdc25C and its principal G2-phase binding partner, 14-3-3. Although S287 phosphorylation is required for 14-3-3 binding, S287 dephosphorylation, which is prohibited by 14-3-3 binding, is not the cause of 14-3-3 release.^32^

Wild-type (WT) or S287A mutant myc-Cdc25Cˆ was expressed in *Xenopus* oocytes via mRNA injection, and proteins extracted from control or progesterone-matured oocytes were incubated with immobilized GST-14-3-3. While GST-14-3-3 efficiently pulled down G2-state myc-Cdc25Cˆ from immature oocytes, it did not pull down the supershifted M-state isoform from mature oocytes. In contrast, GST-14-3-3 failed to pull down the S287A mutant under either condition (Figure 6A). These results recapitulate the S287 phosphorylation-dependent interaction of Cdc25C with 14-3-3 and its release upon M-phase-associated hyperphosphorylation.

**Figure 6:**
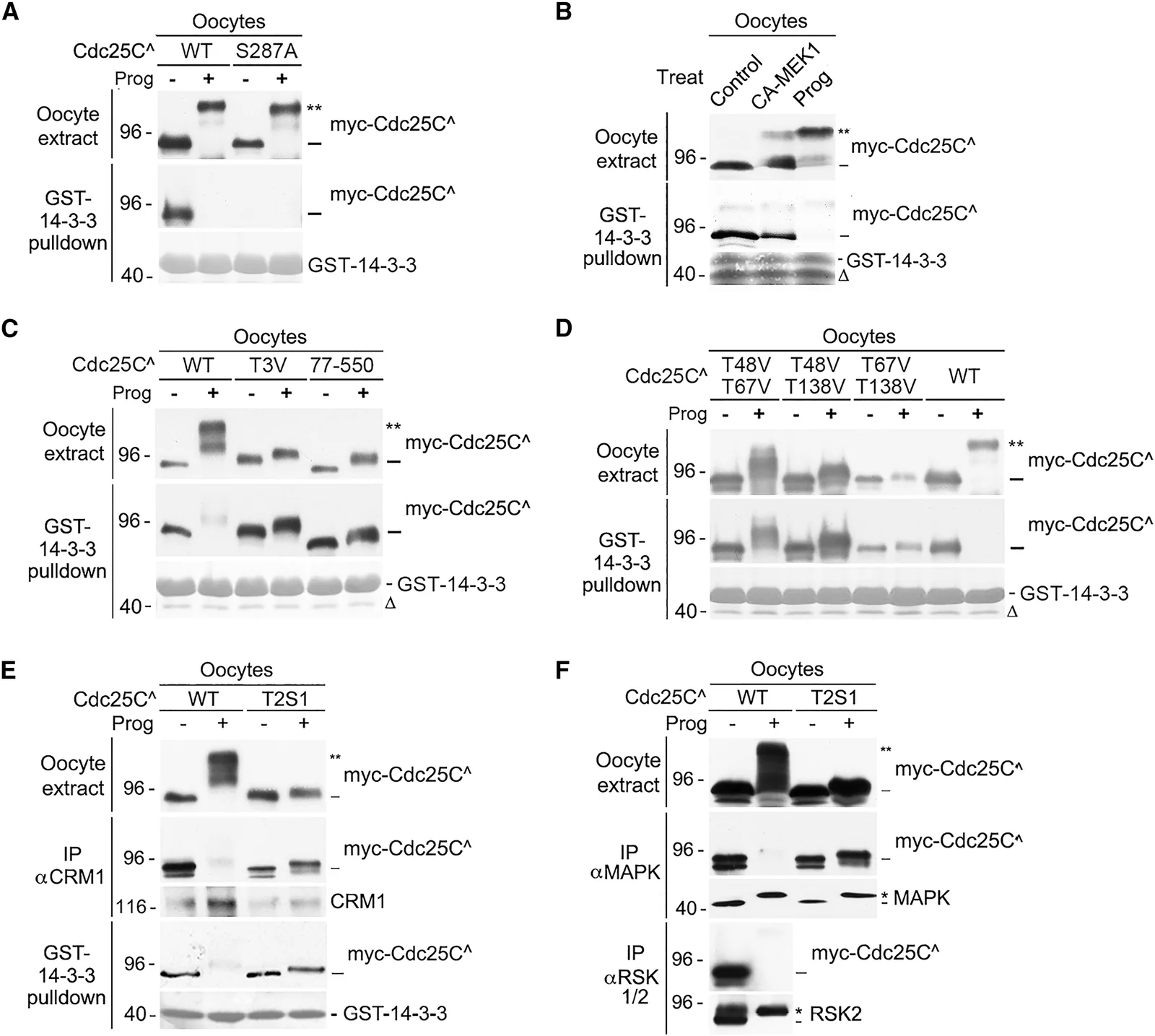
A unique role of site-comprehensive phosphorylation in dissociating G2-state Cdc25C from prebound partners (A) Wild-type (WT) or S287A mutant myc-Cdc25Cˆ was expressed in *Xenopus* oocytes by mRNA injection. Extracts of progesterone-matured (Prog+) or unstimulated (Prog-) oocytes were incubated with immobilized GST-14-3-3. Input oocyte extracts (1/10) and bead eluates were analyzed by anti-Myc immunoblotting after Ponceau S (Ponc S) staining. (B) *Xenopus* oocytes ectopically expressing myc-Cdc25Cˆ were unstimulated (control), injected with a subthreshold level of mRNA encoding myc-CA-MEK1 (CA-MEK1), or matured with progesterone (Prog+). Oocyte extracts were analyzed as in (A). (C) WT and two supershift-defective mutant forms of myc-Cdc25Cˆ were assayed for binding to GST-14-3-3 as in (A). (D) WT and three double phosphodefective mutant forms of myc-Cdc25Cˆ were assayed for binding to GST-14-3-3 as in (A). (E) WT or T48V-T138V-S205A (T2S1) mutant form of myc-Cdc25Cˆ was expressed in *Xenopus* oocytes. Extracts of control or Prog+ oocytes were immunoprecipitated with anti-CRM1 antibodies or incubated with immobilized GST-14-3-3. Input oocyte extracts (1/10) and bead eluates were analyzed by anti-Myc immunoblotting, and bead eluates were also probed with anti-CRM1 antibodies or stained with Ponc S. (F) WT or T2S1 mutant form of myc-Cdc25Cˆ was expressed in *Xenopus* oocytes. Extracts of control or Prog+ oocytes were immunoprecipitated with anti-MAPK or anti-RSK1/2 antibodies. Input oocyte extracts (1/10) and bead eluates were analyzed by anti-Myc immunoblotting, and bead eluates were also immunoblotted with anti-MAPK or anti-RSK2 antibodies.

To determine the respective contributions of the two distinct modes of mitotic phosphorylation to 14-3-3 release, WT myc-Cdc25Cˆ was expressed in oocytes, one-third of which were matured with progesterone and another third co-expressed a subthreshold level of constitutively active MEK1 (CA-MEK1). MEK1-induced activation of the MAPK pathway plays an important role in progesterone-induced oocyte maturation.^33^ While myc-Cdc25Cˆ from progesterone-matured oocytes exhibited a supershift, most myc-Cdc25Cˆ from CA-MEK1-expressing oocytes displayed only a modest mobility shift, consistent with site-specific phosphorylation driven by MAPK activation.^9^ The small supershifted fraction in the CA-MEK1 group likely reflected low-percentage induction of oocyte maturation. Consistent with the electrophoretic mobility patterns, the slightly shifted myc-Cdc25Cˆ was pulled down by GST-14-3-3, whereas the supershifted isoform was not (Figure 6B). These results indicate that only site-comprehensive phosphorylation across the long IDR associates with loss of the interaction of G2-state Cdc25C with 14-3-3.

To further examine this finding, WT and two supershift-deficient mutants of myc-Cdc25Cˆ, T3V (T48V, T67V, and T138V) and 77–550 (lacking the N-terminal region containing T48 and T67), as illustrated in Figure S10A, were expressed in oocytes with or without progesterone stimulation. Consistent with their supershift defects, neither mutant exhibited strong activation upon MEE treatment (Figure S10B), but both mutants from mature oocytes were captured by GST-14-3-3 (Figure 6C). Thus, loss of 14-3-3 binding is associated with site-comprehensive phosphorylation rather than the M-phase cellular environment.

To further investigate the effect of site-comprehensive phosphorylation on 14-3-3 binding, WT and three double phosphodefective mutants of myc-Cdc25Cˆ were expressed in oocytes with or without progesterone stimulation. While the T48V-T138V and T67V-T138V mutants exhibited only minimal mobility shifts upon oocyte maturation, similar to the T3V mutant, the T48V-T67V mutant displayed a ∼15-kDa shift, reflecting a substantially higher extent of phosphorylation. Despite the markedly different extents of phosphorylation, all three mutants from both immature and mature oocytes were pulled down by GST-14-3-3 (Figure 6D), supporting the requirement for site-comprehensive phosphorylation in 14-3-3 release.

We next examined whether site-comprehensive phosphorylation similarly promotes dissociation of other prebound partners, including CRM1, MAPK, and RSK2. Although immobilized GST-Cdc25C_9-374_ did not pull down MAPK or RSK2 from TNT-produced 4K mix (Figure S4D), it readily pulled down endogenous MAPK and RSK2 from IOE (Figure S11A), suggesting that partner interactions within the IDR of G2-state Cdc25C facilitate recruitment of MAPK and RSK2. MAPK and RSK2 binding to G2-state Cdc25C has been mapped to residues 9–287 of the IDR^20^ (Figure S11B), whereas CRM1 docks at two defined sites within the IDR as illustrated in Figure S12A.^18^ WT and supershift-defective T48V-T138V-S205A (T2S1) mutant myc-Cdc25Cˆ^9^^,^^20^ were expressed in oocytes with or without progesterone stimulation, and their interactions with CRM1, MAPK, and RSK2 were assessed by co-immunoprecipitation. Mirroring the 14-3-3 results, WT Cdc25Cˆ associated with CRM1, MAPK, and RSK2 only before oocyte maturation, whereas the T2S1 mutant retained CRM1 and MAPK interactions tested after oocyte maturation (Figures 6E and 6F). These findings indicate that site-comprehensive phosphorylation across the long IDR induces widespread release of prebound partners at M-phase entry.

### Opposing effects of the two distinct types of mitotic phosphorylation on recruitment of cyclin B to G2-state Cdc25C

Beyond its prebound partners, Cdc25C also recruits cyclin B during M-phase induction.^29^ Because cyclin B binding contributes to modest Cdc25C activation before M-phase entry,^27^ we next examined whether site-specific phosphorylation promotes this recruitment. Myc-Cdc25Cˆ was expressed in oocytes that were either matured with progesterone or co-expressed subthreshold CA-MEK1, and its interaction with GST-ΔCycB was assayed by pulldown (Figure 7A). GST-ΔCycB captured very low levels of myc-Cdc25Cˆ from control oocytes but efficiently recovered high levels of the slightly shifted isoform from CA-MEK1-expressing oocytes. From progesterone-matured oocytes, GST-ΔCycB bound high levels of the slightly shifted isoform but very low levels of the supershifted isoform. These results indicate that site-specific phosphorylation via MAPK activation promotes cyclin B recruitment, whereas site-comprehensive phosphorylation at M-phase onset inhibits this interaction.

**Figure 7:**
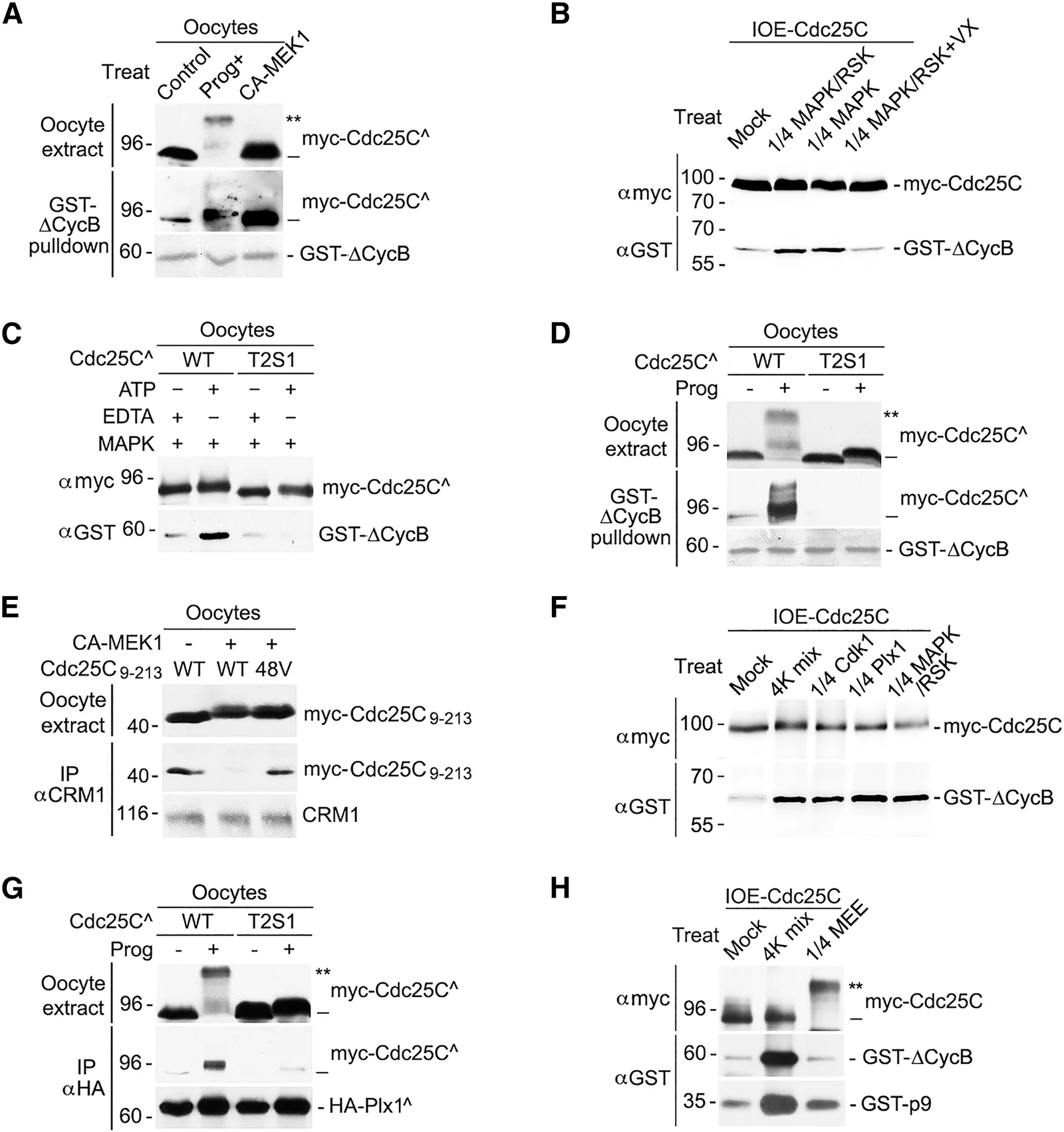
Opposing effects of two distinct types of mitotic phosphorylation on recruitment of new partners to G2-state Cdc25C (A) Oocytes ectopically expressing myc-Cdc25Cˆ were left unstimulated (control), matured with progesterone (Prog+), or injected with a subthreshold level of CA-MEK1 mRNA, and oocyte extracts were incubated with immobilized GST-ΔCycB. Input extracts (1/10) and bead eluates were analyzed by anti-Myc immunoblotting, and bead eluates were also stained with Ponceau S (Ponc S). (B) Myc-Cdc25C immunoprecipitated from HEK293T cell lysates was treated with IOE for 2 h (IOE-Cdc25C) and then mock-treated with EB or incubated with the indicated TNT kinase samples for 2 h. Washed immunocomplexes were incubated with soluble GST-ΔCycB for 1 h, and bead eluates were analyzed by immunoblotting with anti-Myc and anti-GST antibodies. (C) WT or T2S1 mutant myc-Cdc25Cˆ was expressed in *Xenopus* oocytes by mRNA injection, immunoprecipitated with anti-Myc antibodies, and incubated with activated recombinant MAPK in the presence of 1 mM ATP or 10 mM EDTA, which inhibits phosphorylation by chelating magnesium. Washed immunocomplexes were processed as in (B). (D) WT or T2S1 mutant myc-Cdc25Cˆ was expressed in oocytes and analyzed as in (A). (E) WT or T48V mutant myc-Cdc25C_9-213_ was expressed in oocytes with or without co-expression of subthreshold CA-MEK1, and extracts were immunoprecipitated with anti-CRM1 antibodies. Input extracts (1/10) and bead eluates were analyzed by anti-Myc immunoblotting, and bead eluates were also immunoblotted with anti-CRM1 antibodies. (F) IOE-Cdc25C was incubated with the indicated TNT kinase samples for 2 h and then processed as in (B). (G) WT or T2S1 mutant myc-Cdc25Cˆ was co-expressed in oocytes with HA-Plx1ˆ by mRNA injection, and extracts from control or Prog+ oocytes were immunoprecipitated with anti-HA antibodies. Input extracts (1/10) and bead eluates were analyzed by anti-Myc immunoblotting, and bead eluates were also probed with anti-HA antibodies. The results shown are representative of two independent experiments. (H) IOE-Cdc25C was mock-treated with EB or incubated with 1:4-diluted MEE or 4K mix for 2 h, and washed immunocomplexes were incubated with soluble GST-ΔCycB and GST-p9 for 1 h. Bead eluates were analyzed by immunoblotting with anti-Myc and anti-GST antibodies.

To further characterize the role of site-specific phosphorylation, we asked whether MAPK and/or RSK2, the two principal MEK1 downstream kinases during progesterone-induced oocyte maturation, promote cyclin B recruitment. IOE-treated myc-Cdc25C immunoprecipitated from HEK293T cell lysates was phosphorylated with TNT-produced MAPK/RSK2, MAPK alone, or MAPK/RSK2 in the presence of the MAPK inhibitor VX, followed by incubation with soluble GST-ΔCycB. Both MAPK/RSK2 and MAPK alone markedly enhanced GST-ΔCycB binding, whereas MAPK/RSK2 plus VX had no effect (Figure 7B), indicating that MAPK is the responsible kinase. Consistent with this finding, phosphorylation by recombinant MAPK markedly increased GST-ΔCycB binding to WT, but not the MAPK site mutant (T2S1 and T48V-T138V-S205A), myc-Cdc25Cˆ immunoprecipitated from immature oocyte extracts (Figure 7C). Moreover, the T2S1 mutant from either immature or mature oocytes failed to be pulled down by GST-ΔCycB (Figure 7D). Together, these findings indicate that MAPK-mediated phosphorylation within the N-terminal IDR promotes cyclin B recruitment.

To understand how MAPK-mediated phosphorylation promotes cyclin B recruitment, we tested whether it may act by displacing hindering partners. The N-terminal IDR contains potential docking sites for both cyclin B and CRM1 (Figure S12A). Co-expression of CA-MEK1 with myc-Cdc25C_9-213_ markedly reduced CRM1 binding, and this effect was diminished by the phosphodefective T48V mutation in Cdc25C_9-213_ (Figure 7E). Consistent with this result, the phosphomimetic T48D, but not the phosphodefective T48V, mutation diminished CRM1 association (Figure S12B). These findings are consistent with a model in which MAPK promotes cyclin B recruitment through weakening CRM1 binding.

Because ectopic expression of constitutively active Cdk1^20^ or Plx1^34^ can also induce oocyte maturation in the absence of progesterone, we asked whether site-specific phosphorylation by these kinases similarly promotes cyclin B binding. IOE-treated myc-Cdc25C immunoprecipitated from HEK293T cell lysates was phosphorylated with TNT-produced 4K mix or 1:4-diluted Cdk1, Plx1, or MAPK/RSK2, and the treated immunocomplexes were incubated with soluble GST-ΔCycB. Both Cdk1 and Plx1 increased GST-ΔCycB binding to extents similar to those observed with 4K mix or MAPK/RSK2 (Figure 7F), and these effects were sensitive to inhibition of phosphorylation by EDTA (Figure S12C). These results indicate that Cdk1- and Plx1-mediated site-specific phosphorylation can likewise promote cyclin B recruitment to G2-phase Cdc25C, paralleling the MAPK-mediated mechanism.

### Opposing effects of the two distinct types of mitotic phosphorylation on recruitment of Plx1 and CKS/p9 to G2-state Cdc25C

Whereas incubation of immobilized GST-Cdc25C_9-374_ with IOE failed to pull down endogenous Cdk1 or Plx1, incubation with MEE readily did (Figure S13A). Moreover, both TNT-produced cyclin B and Plx1 bound to the N-terminal IDR of Cdc25C in the presence of MEE (Figure S13B). These observations suggested that mitotic phosphorylation of Cdc25C may regulate Plx1 recruitment in a manner similar to that of cyclin B binding. To test this possibility, WT or T2S1 mutant myc-Cdc25Cˆ was co-expressed with HA-tagged catalytically inactive Plx1 (HA-Plx1ˆ)^35^ in *Xenopus* oocytes, and HA-Plx1 was immunoprecipitated from extracts of control or progesterone-matured oocytes (Figure 7G). Consistent with the GST-ΔCycB pulldown results (Figure 7D), little or no myc-Cdc25Cˆ associated with HA-Plx1ˆ in immature oocytes. In mature oocytes, however, a slightly shifted myc-Cdc25Cˆ isoform co-immunoprecipitated with HA-Plx1ˆ, whereas the supershifted isoform did not. Importantly, this M-phase-associated increase in Plx1ˆ binding was abolished by the T2S1 mutation. These results indicate that MAPK-mediated site-specific phosphorylation of G2-state Cdc25C promotes Plx1 binding, whereas site-comprehensive phosphorylation suppresses it, mirroring the regulatory pattern observed for cyclin B binding.

Previous studies showed that phosphorylation of the TP motif at T48 in human Cdc25C promotes its binding to the CKS protein Suc1.^36^ Database analysis (ELM) identified two potential CKS-docking sites in *Xenopus* Cdc25C, flanking the TP motifs at T48 and T112 (Figure S14A). Consistently, immobilized GST-Cdc25C_9-206_ and GST-Cdc25C_9-129_, but not GST-Cdc25C_128-213_, captured TNT-produced myc-p9 in the presence of MEE (Figure S14B), localizing CKS docking to the 9–129 region. However, CKS proteins also bind Cdk1, which could indirectly link CKS to Cdc25C through cyclin B association. We thus assessed the relative contributions of TP phosphorylation and Cdk1 binding by comparing binding of phosphorylated myc-Cdc25C_9-213_ to WT and mutant CKS proteins. While GST-p9 pulled down both Cdk1 and phosphorylated myc-Cdc25C_9-213_, the pTP-binding-defective mutant GST-p9ΔpTP^37^^,^^38^ captured Cdk1 but not phosphorylated myc-Cdc25C_9-213_. In contrast, the Cdk1-binding-defective mutant GST-p9ΔCdk^39^ captured phosphorylated myc-Cdc25C_9-213_ but not Cdk1 (Figure S14C). These results indicate that TP phosphorylation plays a major role in Cdc25C recruitment of CKS proteins.

To compare the effects of site-specific and site-comprehensive phosphorylation on CKS recruitment, IOE-treated myc-Cdc25C immunoprecipitated from TNT products was phosphorylated with either TNT-produced 4K mix or MEE and then incubated with soluble GST-ΔCycB and GST-p9. Mock-treated IOE-Cdc25C bound only low levels of GST-ΔCycB and GST-p9. In comparison, phosphorylation with 4K mix markedly increased binding of both GST-ΔCycB and GST-p9. In contrast, MEE treatment failed to enhance GST-ΔCycB binding and produced only a modest increase in GST-p9 association despite extensive Cdc25C phosphorylation (Figure 7H). These results indicate that site-specific phosphorylation of G2-state Cdc25C promotes CKS recruitment, whereas site-comprehensive phosphorylation markedly limits this effect, further demonstrating the opposing effects of the two phosphorylation modes on new partner recruitment.

## Discussion

Cdc25C is a key regulator of the G2/M transition, yet the mechanistic basis for its phosphoactivation during M-phase induction has remained poorly understood. Our findings identify its long IDR as an interaction hub that mediates intramolecular interactions, substrate recruitment via cyclin B binding, homotypic self-association, and heterotypic interactions with numerous binding partners. This hub exhibits qualitatively distinct responses to site-specific versus site-comprehensive phosphorylation. During M-phase induction, site-specific phosphorylation promotes cyclin B association in association with modest Cdc25C activation before M-phase entry, whereas site-comprehensive phosphorylation disrupts all four classes of IDR-mediated interactions to enable robust Cdc25C activation at M-phase onset. These findings reveal an interaction-based logic of Cdc25C phosphoregulation and link site-comprehensive phosphorylation of a long IDR to large-scale dismantling of its interaction network.

### Overriding effects of site-comprehensive phosphorylation at M-phase onset

Our results demonstrate that site-comprehensive phosphorylation at M-phase onset acts as a dominant molecular switch that overrides the preexisting protein interaction network mediated by the long IDR. Specifically, the extensive modification displaces 14-3-3 despite continued protection of S287 phosphorylation by bound 14-3-3^32^; releases cyclin B and Plx1 without reversing the MAPK-dependent phosphorylations that promote their recruitment (Figures 7A, 7D, and 7G); and dissociates p9 despite the persistence of phosphorylated TP motifs that support p9 binding (Figure 7H). By doing so, the long IDR is no longer regulated through selectively modulating individual binding sites but instead through globally dismantling its interaction network. These overriding effects suggest that site-comprehensive phosphorylation remodels the physicochemical properties of the long IDR so extensively that the IDR becomes broadly incompatible with stable protein-protein interactions.

Each phosphate group contributes two negative charges, and high-density negative-charge accumulation has been shown to substantially expand IDRs by reducing long-range intra-chain contacts.^40^ This electrostatic expansion may change the exposure of hydrophobic residues, thereby diminishing their ability to engage in stable, hydrophobicity-driven interactions. As a result, site-comprehensive phosphorylation may markedly decrease the overall “stickiness” of the long IDR, favoring solvation and permitting only transient, low-affinity contacts. This charge-driven global remodeling provides a mechanistic explanation for how site-comprehensive phosphorylation overrides the interphase interaction network globally. More broadly, because site-comprehensive phosphorylation of long IDRs underlies the M-phase supershift of numerous regulatory and structural proteins,^5^ this mode of phosphoregulation may represent a general mechanism by which cells rapidly and broadly dismantle preexisting protein interaction networks mediated by long IDRs.

### Functional switching by site-comprehensive phosphorylation

The IDR of Cdc25C, comprising nearly two-thirds of the protein, performs numerous non-catalytic functions through an extensive network of protein-protein interactions (Figures S1 and S2). In *Xenopus*, for example, Cdc25C binding to 14-3-3 promotes cytoplasmic retention, interaction with CRM1 drives nuclear export, and association with importin contributes to nuclear import.^17^^,^^18^ In human cells, Cdc25C is enriched in nuclear speckles^41^ (Figure S15), dynamic membraneless subnuclear structures involved in pre-mRNA processing.^42^ These diverse interphase functions highlight a dual functional architecture: IDR-mediated interaction roles and Cdk1 dephosphorylation, which requires coordinated contributions from both the regulatory and catalytic domains. From this perspective, the site-comprehensive phosphorylation of the IDR at M-phase onset does more than activate Cdc25C: it enforces a transition from the IDR’s interaction-based roles to a catalytically active phosphatase.

As one of the many phosphoproteins that undergo the M-phase supershift, Cdc25C may represent a broader class of long-IDR-containing proteins that undergo functional repurposing through site-comprehensive phosphorylation at M-phase entry. Ki-67 is one such example. During interphase, its long IDR associates with nucleolar chromatin and the nuclear matrix, where it contributes to heterochromatin organization and ribosome biogenesis. Coincident with site-comprehensive phosphorylation of its long IDR at M-phase entry, Ki-67 dissociates from these interphase structures and redistributes to the surface of condensed chromosomes to form the perichromosomal layer,^43^ where it prevents chromosome clustering and maintains chromosome individuality.^44^ Thus, site-comprehensive phosphorylation at M-phase entry may constitute a general mechanism for coordinately reorganizing subcellular architecture and repurposing numerous proteins from their interphase-specific functions to mitosis-specific activities.

### Determinants of Cdc25C activity

Although robust activation of Cdc25C by site-comprehensive phosphorylation is associated with disruption of all four classes of IDR-mediated interaction, these interaction classes may contribute differently to Cdc25C activity. Among the four interaction classes, the IDR-catalytic domain interaction appears to be the weakest, as it was disrupted by G2-state conversion, site-specific phosphorylation, and the 62DE mutation. Importantly, these different perturbations had markedly different effects on Cdc25C activity, making it unlikely that the IDR-catalytic domain interaction constitutes a critical determinant of Cdc25C activity. This interpretation is consistent with previous observations that full-length human Cdc25C and its isolated catalytic domain exhibited similar basal phosphatase activities.^26^

Unlike the IDR-catalytic domain interaction, cyclin B association was preserved following site-specific phosphorylation and the 62DE mutation but markedly reduced during G2-state conversion in association with decreased basal activity. Conversely, site-specific phosphorylation before M-phase entry enhanced cyclin B association concomitant with modest Cdc25C activation, although robust activation at M-phase onset occurred with little cyclin B binding. Consistent with previous observations that disruption of the Cdc25C-cyclin B interaction specifically impairs the modest activation of Cdc25C before M-phase entry but has little effect on robust activation within M-phase,^27^ these findings support a model in which substrate recruitment through cyclin B binding specifically promotes the early, modest activation of Cdc25C.

In contrast to both the IDR-catalytic domain interaction and cyclin B association, the IDR-mediated self-association of Cdc25C was resistant to G2-state conversion, the 62DE mutation, and partial site-comprehensive phosphorylation, and was efficiently abolished only upon completion of site-comprehensive phosphorylation. These findings indicate that intrinsically strong self-association may constitute an autoinhibitory mechanism that is relieved only during robust activation by site-comprehensive phosphorylation.

Distinct from the other interaction classes, heterotypic interactions involve a large number of binding partners that collectively form an interaction network. While no individual heterotypic interaction has been consistently found to affect Cdc25C activity, their collective presence during G2-state conversion correlated with reduced basal activity and an increased threshold for phosphoactivation (Figure 1). Thus, the heterotypic interaction network may be viewed as a collective inhibitory mechanism that imposes physical constraints on Cdc25C activity, and site-comprehensive phosphorylation may constitute the key mechanism that globally relieves these constraints.

### Biphasic regulation of Cdc25C activity during M-phase induction

Having identified the three classes of IDR-mediated interactions likely to affect Cdc25C activity, the remaining question is how these mechanisms may act in concert to produce its biphasic regulation during M-phase induction. Based on the findings in this study, newly synthesized original-state Cdc25C readily associates with cyclin B, and therefore possesses appreciable basal activity despite the presence of strong self-association. Because G2-state Cdc25C becomes incorporated into an interaction network, it may possess reduced basal activity through inhibition of cyclin B binding and an increased threshold for phosphoactivation due to the need to disrupt this network by site-comprehensive phosphorylation. However, the site-comprehensive phosphorylation program is tightly controlled in interphase cells, and its M-phase-specific activation likely requires a threshold level of active Cdk1. To reach this threshold, site-specific phosphorylation of G2-state Cdc25C by upstream signaling kinases restores cyclin B association without dismantling the inhibitory interaction network, thereby only modestly stimulating Cdc25C activity. This modest activation helps generate the threshold level of active Cdk1 needed to activate the site-comprehensive phosphorylation program. Once activated, site-comprehensive phosphorylation globally dismantles the interaction network mediated by the Cdc25C IDR, thereby relieving the steric and topological constraints that limit Cdc25C activity. In this fully activated state, stable cyclin B association is no longer required for efficient substrate recognition by Cdc25C. Thus, the biphasic regulation of Cdc25C may reflect two mechanistically distinct phases: an initial regulatory phase in which a threshold level of Cdc25C activity triggers the site-comprehensive phosphorylation program, followed by an execution phase that converts Cdc25C into its fully active mitotic state. More broadly, this two-phase regulatory logic illustrates how cells can use site-specific phosphorylation to trigger site-comprehensive phosphorylation, thereby driving an abrupt and decisive system transition.

### Limitations of the study

Although we provide correlative evidence that robust activation of Cdc25C by site-comprehensive phosphorylation is associated with disruption of both IDR-mediated self-association and heterotypic interactions with numerous binding partners, establishing a causal relationship will require a means to selectively disrupt these interactions. In addition, because Cdc25C activity in this study was assessed by monitoring a decrease in Cdk1 inhibitory phosphorylation at Tyr15 using phospho-specific immunoblotting, the substrate could not be supplied in large excess as is typically done in standard enzymatic assays. Consequently, the Cdc25C activity assays provide primarily qualitative, rather than quantitative, information regarding the relative contributions of the two phosphorylation modes to Cdc25C activation.

## Resource availability

### Lead contact

Further information and requests for resources and reagents should be directed to and will be fulfilled by the lead contact, Dr. Jian Kuang (jkuang@mdanderson.org).

### Materials availability

Plasmids generated in this study are available from the lead contact with a completed materials transfer agreement.

### Data and code availability

• Full, uncropped immunoblots for Figures 1, 2, 3, 4, 5, 6, and 7 have been deposited in Mendeley Data and are publicly accessible at Mendeley Data: https://doi.org/10.17632/k3pn453rvp.1. The DOI is also provided in the key resources table.
• This paper does not report original code.
• Additional information on data reported in this paper is available from the lead contact upon request.

## Acknowledgments

This work was supported by a 10.13039/100000005DOD/10.13039/100014039PCRP grant (W81XWH-09-1-0274) and an IRG grant awarded to J.K., 10.13039/100000002NIH grants (CA174798) and 10.13039/100004917CPRIT grants (RP150179 and RP150282) awarded to S.-H.L., and a grant from the 10.13039/501100004735Natural Science Foundation of Hunan Province (2020JJ5478) awarded to T.T. The Proteomics and Metabolomics Facility was supported in part by 10.13039/100004917CPRIT (RP130397) and 10.13039/100000002NIH (1S10OD012304-01 and P30CA016672). The DNA Analysis Facility at The University of Texas MD Anderson Cancer Center was supported by 10.13039/100000002NIH/10.13039/100000054NCI (P30CA016672).

## Author contributions

T.T. designed and performed most experiments and contributed to manuscript preparation. C.W. assisted with study design, optimized several experimental conditions, and performed several key experiments. R.W. designed and carried out most of the oocyte experiments shown in Figure 7. W.Z. generated critical reagents. S.-H.L. contributed to study design, data analysis, and manuscript preparation. J.K. conceived and supervised the project and wrote the manuscript.

## Declaration of interests

The authors declare no competing interests.

## STAR★Methods

### Key resources table

**Table:** 

REAGENT or RESOURCE	SOURCE	IDENTIFIER
**Antibodies**		

anti-Myc for immunoblot (IB)	MilliporeSigma	Cat# SAB4301136
anti-Myc for immunoprecipitation (IP)	Cell Signaling Technology	Cat# 2276; RRID: AB_331783
anti-Myc for IB	Santa Cruz Biotechnology	Cat# sc-40; RRID: AB_627268
Anti-Myc for IB or IP	OriGene	Cat# TA150121; RRID: AB_2622266
anti-HA tag for IB or IP	Santa Cruz Biotechnology	Cat# sc-805; RRID: AB_631618
anti-Cdk1 pY15 for IB	Cell Signaling Technology	Cat# 9111; RRID: AB_331460
anti-Cdk1 for IB	Santa Cruz Biotechnology	Cat# sc-54; RRID: AB_627224
anti-pT202/pY204 ERK1/2 for IB	Cell Signaling Technology	Cat# 9106; RRID: AB_331768
anti-Xenopus MAPK for IP or IB	Our 2007 paper^20^	N/A
anti-ERK2 for IB	Santa Cruz Biotechnology	Cat# sc-1647; RRID: AB_627547
Anti-RSK1/2 for IP	Cell Signaling Technology	Cat# 9355; RRID: AB_659900
anti-RSK2 for IB	Santa Cruz Biotechnology	Cat# sc-9986; RRID: AB_672176
anti-Plk1 for IB	Invitrogen	Cat# 33-1700; RRID: AB_2533104
anti-CRM1 for IB or IP	Santa Cruz Biotechnology	Cat# sc-74454; RRID: AB_1122704
anti-GST for IB	Santa Cruz Biotechnology	Cat# sc-459; RRID: AB_631586
anti-GST for IB	HUABIO	Cat# ET1611-47; RRID: AB_3070026
anti-K/HpSP for IB	Cell Signaling Technology	Cat# 9477; RRID: AB_2714143
anti-RXXpS/T for IB	Cell Signaling Technology	Cat# 9614; RRID: AB_331810
anti-phospho-Histone H1 for IB	Upstate Biotechnology	Cat# 06-597
HRP-conjugated goat anti-mouse IgG	Bio-Rad	Cat# 1706516; RRID: AB_2921252
HRP-conjugated goat anti-rabbit IgG	Bio-Rad	Cat# 1706515; RRID: AB_11125142

**Bacterial and virus strains**		

Competent BL21 E. coli	Thermo Fisher Scientific	Cat# EC0114

**Chemicals, peptides, and recombinant proteins**		

Cdk1/2 inhibitor RS	LC Laboratories	Cat# R1234
ERK inhibitor VX-11e	ChemieTeK	Cat# 896720
RSK inhibitor SL0101	MedChemExpress	Cat# HY-15237
Plk1 inhibitor BI2536	Selleck Chemicals	Cat# S1109
staurosporine	MilliporeSigma	Cat# S4400
PKA inhibitor	BACHEM	Cat# H-3222
PKA inhibitor H-89	Beyotime	Cat# S1643
reduced glutathione	Sigma-Aldrich	Cat# 3541
Glutathione Sepharose	Solarbio Life Sciences	Cat# P2020
Protein A/G agarose	Thermo Fisher Scientific	Cat# 20421
Q-sepharose	Pharmacia	Cat# 17-0510-01
Ni-NTA agarose	GE Healthcare Life Sciences	Cat# 17-5268-01
ATP	Solarbio Life Sciences	Cat# A8270
ATP-γ-S	Roche	Cat# 11162306001
okadaic acid (OA)	Calbiochem	Cat# 495604
Microcystin (MC)	Cayman Chemical Company	Cat# 10007188
protease inhibitor cocktail	Roche	Cat# 11697498001
calf thymus histone H1	MilliporeSigma	Cat# 382150
Dulbecco’s modified Eagle’s medium	Gibco	Cat# C11995500BT
fetal bovine serum	Cellmax	Cat# SA301.02.V
L-glutamine	Solarbio Life Sciences	Cat# G8230
penicillin and streptomycin	Solarbio Life Sciences	Cat# P1400
nocodazole	Sigma-Aldrich	Cat# M1404
Ponceau S	Sigma-Aldrich	Cat# P7170
Prestained molecular weight standards	MilliporeSigma	Cat# SDS7B2
Prestained molecular weight standards	Thermo Scientific	Cat# 26616
human chorionic gonadotropin	MilliporeSigma	Cas# 9002-61-3
Progesterone	Sigma-Aldrich	Cat# 5341

**Critical commercial assays**		

TnT® Quick Coupled Transcription/Translation System	Promega	Cat# L2080
mMESSAGE mMACHINE™ High-Yield Capped RNA Transcription Kit	Thermo Fisher Scientific	Cat# AM1340
site-directed mutagenesis kit	TransGen Biotech	Cat# FM1-11
PCR polymerase	Takara	Cat# R050A

**Deposited data**		

Full, uncropped immunoblots for Figures 1, 2, 3, 4, 5, 6, and 7	Mendeley Data	https://doi.org/10.17632/k3pn453rvp.1

**Experimental models: Cell Lines**		

HEK293T	Procell	Cat# CL-0005

**Experimental models: Organisms/strains**		

*Xenopus laevis*	Xenopus express	Cat# 470218-402

**Recombinant DNA**		

pCS2+MT-Cdc25C	Our 2007 paper^20^	N/A
pCS2+MT-Cdc25C C457S (Cdc25Cˆ)	Our 2007 paper^20^	N/A
pCS2+HA-Cdc25C C457S (Cdc25Cˆ)	Our 2007 paper^20^	N/A
pCS2+MT-Cdc25C 9-375	Our 2022 paper^5^	N/A
pCS2+HA-Cdc25C 9-375	this paper	N/A
pCS2+MT-Cdc25C 9–375 62DE	Our 2022 paper^5^	N/A
pCS2+MT-Cdc25C 62DE	Our 2025 paper^9^	N/A
pCS2+MT-Cdc25C S287A	this paper	N/A
pCS2+MT-Cdc25Cˆ S287A	this paper	N/A
pCS2+MT-Cdc25C 375-550	Our 2007 paper^20^	N/A
pCS2+MT-Cdc25C 9-129	Our 2022 paper^5^	N/A
pCS2+MT-Cdc25C 9-213	Our 2007 paper^20^	N/A
pCS2+MT-Cdc25C 9–213 T48V	this paper	N/A
pCS2+MT-Cdc25C 9–213 T48D	this paper	N/A
pCS2+MT-Cdc25C 9-287	Our 2007 paper^20^	N/A
pCS2+MT-Cdc25C 205-550	Our 2022 paper^5^	N/A
pCS2+MT-Cdc25C 126-213	this paper	N/A
pCS2+MT-Cdc25C 212-375	this paper	N/A
pCS2+MT-Cdc25C 77-550	Our 2022 paper^5^	N/A
pCS2+MT-Cdc25C 77–550ˆ	this paper	N/A
pCS2+MT-Cdc25CT48V-T67V-T138V (T3V)	Our 2025 paper^9^	N/A
pCS2+MT-Cdc25Cˆ T3V	Our 2025 paper^9^	N/A
pCS2+MT-Cdc25Cˆ T48V-T67V	Our 2025 paper^9^	N/A
pCS2+MT-Cdc25Cˆ T48V-T138V	Our 2025 paper^9^	N/A
pCS2+MT-Cdc25Cˆ T138V-T67V	Our 2025 paper^9^	N/A
pCS2+MT-Cdc25CˆT48V-T138V-S205A (T2S1)	Our 2007 paper^20^	N/A
pCS2+MT-Gwl 179-745	Our 2022 paper^5^	N/A
pCS2+MT-Wee1A 1-199	Our 2022 paper^5^	N/A
pCS2+MT-Cdk1 T161E	Our 2022 paper^5^	N/A
pCS2+MT-sea urchin cyclin B Δ90	Our 2022 paper^5^	N/A
pCS2+MT-p9	Our 2022 paper^5^	N/A
pCS2+MT-CA-MEK1	Our 2007 paper^20^	N/A
pCS2+HA-CA-MEK1	Our 2022 paper^5^	N/A
pCS2+HA-Plx1	this paper	N/A
pCS2+HA-CA-Plx1	Our 2022 paper^5^	N/A
pCS2+HA-Plx1 K82R (Plx1ˆ)	this paper	N/A
pCS2+MT-MAPK	this paper	N/A
pCS2+HA-MAPK	Our 2022 paper^5^	N/A
pCS2+HA-RSK2	Our 2022 paper^5^	N/A
pGEX6P1-p9	Our 2022 paper^5^	N/A
pGEX6P1-p9ΔCdk1(E63Q)	this paper	N/A
pGEX6P1-p9ΔpTP (K11E-S51E-R71A)	this paper	N/A
pGEX4T3-Cdc25Cˆ 375-550	Our 2025 pape^9^	N/A
pGEX4T3-Cdc25C 205-375	this paper	N/A
pGEX4T3-Cdc25Cˆ 205-550	Our 2010 paper^45^	N/A
pGEX4T3-Cdc25C 9-374	Our 2022 paper^5^	N/A
pGEX4T3-Cyclin B2	this paper	N/A
pGEX4T3-sea urchin cyclin B Δ90	Our 1994 paper^46^	N/A
pGEX4T3-Cdc25C 9-206	Our 2010 paper^45^	N/A
pGEX4T3-Cdc25C 9-129	Our 2007 paper^20^	N/A
pGEX4T3-Cdc25C 128-213	Our 2007 paper^20^	N/A
pET28a(+)-Cdc25C-EGFP-His	this paper	N/A

**Software and algorithms**		

ImageJ (version 1.44p)	National Institutes of Health	Version 1.44p
MetaMorph (version 7.7.5.0)	Molecular Devices	Version 7.7.5.0

### Experimental model and study participant details

#### *Xenopus laevis*

Adult female *Xenopus laevis* were used in this study to obtain unfertilized eggs and immature oocytes. To obtain unfertilized eggs, adult female frogs were injected with 800 IU of human chorionic gonadotropin to induce ovulation. The ovulated unfertilized eggs were first dejelled with 2% cysteine and then homogenized in an EB-based extraction buffer to prepare MEE. To obtain immature oocytes, ovarian tissues were surgically removed from adult female frogs under anesthesia. From acquired ovarian tissues, fully grown oocytes were obtained either through manual defolliculation under a dissecting microscope or through treatment with 0.2% collagenase at 22°C for 1-3 hr. Oocytes acquired from manual defolliculation were used in microinjection of *in vitro* transcribed mRNA and time-course examination of protein phosphorylation in progesterone-induced oocyte maturation. Oocytes acquired from collagenase treatment were homogenized in an EB-based extraction buffer to prepare IOE.

All animal procedures were approved by the Institutional Animal Care and Use Committee (IACUC) of The University of Texas MD Anderson Cancer Center (Protocol #: 00000396-RN02) and conducted in accordance with applicable institutional and national guidelines for the care and use of laboratory animals.

#### Cell culture

HEK293T cells were obtained from Procell (Cat# CL-0005) and authenticated by the supplier using short tandem repeat (STR) profiling. Cells were cultured in 60-mm cell culture dishes in Dulbecco’s modified Eagle’s medium (DMEM) supplemented with 10% fetal bovine serum, 2 mM L-glutamine, and 1% penicillin/streptomycin at 37°C in a cell culture incubator with 5% CO_2_. Cell growth was observed under an optical microscope, and confluent cultures were subcultured 1:3 by trypsinization per standard protocols. Mycoplasma testing was not performed in this study.

### Method details

#### Large-scale preparation of MEE and IOE

M phase-stabilized *Xenopus* egg extracts (MEE) were prepared from unfertilized *Xenopus laevis* eggs according to Wu and Gerhart^47^ with minor modifications. Briefly, female frogs were injected with 800 IU human chorionic gonadotropin (MilliporeSigma; 9002-61-3) to induce ovulation approximately 12 h before egg collection. Eggs were dejellied in 2% cysteine prepared in 100 mM Tris-HCl (pH 7.8) and washed extensively with Modified Barth’s Solution (MBS) containing 88 mM NaCl, l mM KCl, 2.4 mM NaHCO_3_, 0.82 mM MgSO_4_, 0.33 mM Ca(NO3)_2_, 0.41 mM CaCl_2_,10 mM Hepes (pH7.4). Dejellied eggs were packed in egg extraction buffer (EB)^47^ containing 80 mM sodium β-glycerophosphate, 20 mM EGTA, and 15 mM MgCl_2_ (pH 7.3), and homogenized by pipetting with an equal volume of EB supplemented with 5 mM dithiothreitol (DTT), 20 mM NaF, 1 mM ATP-γ-S (Roche; 11162306001), 1 μM okadaic acid (OA; Calbiochem; 495604), and 10 μg/mL each of leupeptin (Roche; 11017101001), chymostatin (Roche; 11004638001), and pepstatin (Roche; 11359053001). The homogenate was centrifuged at 160,000 × g for 1 h at 4°C, and the translucent cytoplasmic layer between the lipid cap and pellet was collected as MEE.

Immature *Xenopus* oocytes were obtained by collagenase treatment of surgically excised ovarian tissue from female frogs. Immature oocyte extracts (IOE) were prepared as described above for MEE except that ATP-γ-S and OA were omitted from the extraction buffer. The protein concentrations of both MEE and IOE were typically 8-10 mg/mL. Extracts were stored at -80°C until use.

#### Preparation of GST and GST-tagged proteins

The pGEX6P1-based expression plasmid for GST-p9 was described previously.^5^ The expression constructs for GST-tagged p9ΔCdk1(E63Q) and p9ΔpTP (K11E-S51E-R71A) were generated by site-directed mutagenesis (TransGen Biotech; FM111). pGEX4T3-based expression plasmids for GST, GST-tagged Cdc25C proteins, and GST-tagged cyclin proteins were either reported previously^5^^,^^9^^,^^20^^,^^45^^,^^46^ or newly generated by PCR amplification from validated cDNAs followed by subcloning into pGEX4T3. *Xenopus* cyclin B2 cDNA^48^ was provided by Dr. Hideki Kobayashi. Bacterial expression constructs for GST-tagged human 14-3-3θ and 14-3-3η were kindly provided by Dr. Mong-Hong Lee (MD Anderson Cancer Center).

GST fusion proteins were expressed in *E. coli* BL21 cells and affinity-purified onto Glutathione-Sepharose (Solarbio Life Sciences; P2020) as described previously.^5^ Immobilized proteins (typically 0.3-3 μg/μL) were stored in PBS at 4°C for ≤1 week. Soluble GST fusion proteins were obtained by elution with EB containing 10 mM reduced glutathione, 1 mM DTT, and 1× protease inhibitor cocktail (Roche; 11697498001) at 22°C for 15 min.

#### Preparation of p9-Cdk1

Fifty microliters of GST-p9 beads were incubated with 500 μL of MEE at 4°C for 2 h. The beads were washed three times with TBS (50 mM Tris-HCl, pH 7.4, 150 mM NaCl) supplemented with 0.1% Triton X-100, followed by two washes with TBS alone. Bound proteins were eluted in 100 μL of TBS supplemented with 10 mM reduced glutathione, 1 mM DTT, and 1× protease inhibitor cocktail at 22°C for 15 min. The eluate, designated p9-Cdk1, retained histone H1 kinase activity for up to 5 days when stored at 4°C. Immediately before use in phosphorylation reactions, p9-Cdk1 was supplemented with 5 mM β-glycerophosphate, 10 mM MgCl_2_, 1 mM ATP, and 1 μM OA or MC.

#### *In vitro* transcription-linked translation

pSC2+MT-based expression plasmids for myc-tagged proteins were either described previously^5^^,^^9^^,^^20^ or newly generated by PCR amplification from validated cDNAs followed by subcloning into the pSC2+MT vector. pSC2+HA-based expression plasmids for HA-tagged proteins were either described previously^5^^,^^9^ or newly generated by PCR amplification from validated cDNAs followed by subcloning into the pSC2+HA vector. Other pCS2+MT- or pCS2+HA-based expression plasmids encoding myc-tagged or HA-tagged mitotic kinases and kinase components were described previously.^5^

*In vitro* transcription-linked translation (TNT) was performed using the TnT® Quick Coupled Transcription/Translation System (Promega; L2080) according to the manufacturer’s instructions. TNT products were used immediately or stored in small aliquots at -80°C until use.

#### Preparation of major mitotic kinases by TNT

Active kinases were freshly prepared from TNT products in 1× phosphorylation buffer (50 mM Tris-HCl pH 7.5, 10 mM MgCl_2_, 5 mM β-glycerophosphate, 1 mM ATP, 1 mM DTT, 1 μM MC, and 1× protease inhibitor cocktail) as reported previously^5^ and detailed below:

• **Cdk1 complex**: Myc–Cdk1 T161E (2 μL), ΔCyclin B (2 μL), and *Xenopus* p9 (1 μL) were mixed with 1 μL 6× phosphorylation buffer at 22°C for 30 min. The resulting product exhibited histone H1 kinase activity comparable to that of MEE (Figure S4B).
• **Plx1**: HA-CA-Plx1 (1 μL) was mixed with 1 μL 2× phosphorylation buffer at 22°C for 30 min, yielding a slightly higher level of Plx1 protein than that of MEE.
• **MAPK/RSK2 mixture**: HA-CA-MEK (0.5 μL), HA-MAPK (1 μL), and HA-RSK2 (3.5 μL) were mixed with 1 μL 6× phosphorylation buffer and incubated at 22°C for 1 h, producing comparable levels of active MAPK and RSK2 to MEE.
• **MAPK alone**: HA-CA-MEK (0.5 μL) and HA-MAPK (1 μL) were mixed with 1 μL 6× phosphorylation buffer and 3.5 μL H_2_O, yielding active MAPK at levels comparable to those in MEE (Figure S4C).
• **Four-kinase mixture**: Equal volumes of the TNT-produced Cdk1 complex, MAPK/RSK2 mixture, and Plx1 preparations were combined with one volume of 1× phosphorylation buffer, resulting in a fourfold dilution of each kinase relative to the individual preparations.

To suppress intrinsic PKA activity present in TNT lysates, 100 μM PKA inhibitor (BACHEM; H-3222) or 50 μM H-89 (Beyotime; S1643) was routinely added before phosphorylation reactions.

#### Kinase inhibition in MEE

The Cdk1/2 inhibitor Roscovitine (RS; LC Laboratories; R1234) was added to MEE at 10 μM. The ERK inhibitor Vx-11e (VX; ChemieTeK; 896720) was added at 0.1 μM, the RSK inhibitor SL0101 (SL; MedChemExpress; HY-15237) at 50 μM, the Plk1 inhibitor BI2536 (BI; Selleck Chemicals; S1109) at 2 μM, and the pan-kinase inhibitor staurosporine (ST; MilliporeSigma; S4400) at 25 μM. All inhibitors were dissolved in DMSO, and stock solutions were stored in small aliquots at -80°C until use. For inhibition assays, MEE was preincubated on ice with DMSO or the indicated kinase inhibitors at the specified concentrations for 3-5 min before phosphorylation of substrate proteins.

#### Cdc25C activity assays

The workflow for Cdc25C activity assays is outlined in Figure S3A. To prepare IP-Cdc25C, 5 μL of TNT-produced myc-Cdc25C was incubated with 1 μL anti-Myc antibody (Cell Signaling Technology; 2276) and 3-5 μL protein A/G agarose (Thermo Fisher Scientific; 20421) at 22°C for 1 h or at 4°C overnight. The beads were washed three times with 200 μL EB. To prepare IOE-Cdc25C, IP-Cdc25C beads were incubated with IOE for 1 h and then washed three times with EB. For phosphorylation, IP-Cdc25C or IOE-Cdc25C beads were incubated with 10 μL of kinase sample or EB alone at 22°C for 1 or 2 h, washed three times with EB, and rinsed once with 15 μL of EB supplemented with 1 mM ATP, 1 mM DTT, and 1 μM microcystin (MC). For Cdc25C activity assays, the treated beads were incubated with substrate samples at 22°C for the indicated times. At each time point, the beads were briefly pelleted, and 20 μL of the supernatant was mixed immediately with 10 μL of 5× SDS-PAGE sample buffer. Aliquots (15 μL) were analyzed by immunoblotting with antibodies against total Cdk1 or Y15-phosphorylated Cdk1.

#### Preparation of substrates for Cdc25C activity assays

For Figures 1D and S10B, the substrate was a 1:10 dilution of high-quality IOE supplemented with 1 μM OA (IOE∗/OA). IOE∗ was prepared from manually defolliculated stage VI *Xenopus* oocytes by homogenization in an equal volume of EB containing 5 mM DTT and protease inhibitors, followed by centrifugation at >400,000 × g at 4°C for 1 h. The translucent cytoplasmic layer between the lipid cap and yolk platelets was collected and stored at -80°C.

For Figures 1B, 1C, and 2, the substrate was partially purified phosphorylated Cdk1 (pCdk1), prepared as outlined in Figure S3B. Briefly, M-phase egg extract (MEE) was subjected to sequential Q-Sepharose chromatography to enrich endogenous Cdk1. Cdk1-peak fractions were identified by immunoblotting, pooled as QF-Cdk1, verified for histone H1 kinase activity, aliquoted, and stored at −80°C. To generate pCdk1, myc-Wee1A and myc-Myt1 were co-immunoprecipitated and incubated with QF-Cdk1 supplemented with 1 mM ATP, 1 μM OA or MC, 1 mM DTT, and protease inhibitor cocktail at 22°C for 1 h. Following brief centrifugation, the supernatant was collected as pCdk1 and used in subsequent Cdc25C activity assays.

#### GST pulldown of myc-tagged Cdc25C proteins and mitotic kinases

To examine intramolecular interactions of original-state Cdc25C or its binding to *Xenopus* cyclin B2, 2 μL of TNT-produced myc-Cdc25C protein was mixed with 10 μl EB supplemented with 1 mM DTT and 1× protease inhibitor cocktail, and incubated with 5 μl immobilized GST-Cdc25C_375–550ˆ_ or GST-CycB2 (GST-tagged *Xenopus* cyclin B2) at 22°C for 90 min. The beads were washed twice with EB, and bound proteins were eluted with SDS-PAGE sample buffer. Gel-separated proteins were stained with Ponceau S and analyzed by immunoblotting with anti-myc antibodies. GST-pulldown assays of myc-tagged Gwl and Wee1A proteins were performed similarly.

To examine the effects of phosphorylation, 2 μL of TNT-produced myc-Cdc25C was first incubated with 4 μL of kinase samples at 22°C for 2 h, diluted 1:1 with 2× EB, and then subjected to GST pulldown assays as described above.

To characterize interactions between Cdc25C and mitotic kinases, 20 μL of TNT-produced four-kinase mixture (4K mix), IOE, or MEE was incubated with 5-10 μL immobilized GST-Cdc25C_9-374_ or GST-Cdc25C_375-550ˆ_ at 22°C for 15 min. After two washes with EB, bound proteins were analyzed by immunoblotting with the indicated antibodies following Ponceau S staining.

#### Culture, transfection, and extraction of HEK293T cells

HEK293T cells were cultured, transfected, harvested, and extracted as described previously.^9^ Immunoprecipitation of overexpressed myc-tagged Cdc25C proteins from HEK293T cell lysates and subsequent treatment of the resulting immunocomplexes with IOE were performed as detailed in the Cdc25C activity assays. Unlike TNT-produced myc-Cdc25C or myc-Cdc25C_9-375_, myc-tagged Cdc25C proteins overexpressed in HEK293T cells exhibited little or no detectable truncation products.

#### Cdc25C self-association assays

For Figure 5A, 5 μL of TNT-produced myc-Cdc25C_9–375_ was immunoprecipitated with 1 μL of anti-Myc antibody (OriGene; TA150121) and 3-5 μL of protein A/G agarose beads for 2 h at 22°C. After three washes with 200 μL of EB, the immunocomplex was incubated with 5 μL of TNT-produced HA-Cdc25C_9–375_ in 20 μL of EB supplemented with 1 mM DTT and protease inhibitor cocktail for 1 h at 22°C. The beads were then washed twice with 200 μL of the indicated wash buffer, and bound proteins were eluted with 10 μL of 1.5× SDS sample buffer. Bead eluates were analyzed by immunoblotting with anti-HA and anti-Myc antibodies.

For Figures 5B–5D, 5 μL of TNT-produced myc-Cdc25C_9–375_ were immunoprecipitated as described above. The resultant immunocomplex and soluble HA-Cdc25C_9–375_ were separately treated with EB, IOE, MEE or ME at 22°C for the indicated times. After washing, the immunocomplex was incubated with the correspondingly treated soluble protein in 20 μL of EB supplemented with 1 mM DTT and protease inhibitor cocktail for 1 h at 22°C. The beads were then processed as described above. Where indicated, 2 μL of TNT-produced myc-Cdc25C_9–375_ 62DE was immunoprecipitated and incubated with 2 μL of TNT-produced soluble HA-Cdc25C_9–375_ 62DE without prior treatment.

For Figures S8A and S8B, myc- and HA-tagged versions of the same protein were coproduced by TNT, and 5 μL of TNT products were immunoprecipitated with 1 μL of anti-Myc antibody and 3-5 μL of protein A/G agarose beads for 2 h at 22°C. After washing with the indicated wash buffer, the beads were processed as described above.

#### Fluorescence imaging of GFP-tagged Cdc25C proteins

The EGFP coding sequence was amplified by PCR from the pEGFP-C3 vector and inserted into the pCS2+MT-Cdc25C construct between the XhoI and XbaI sites, placing EGFP at the C terminus of Cdc25C. The resulting Cdc25C-EGFP coding sequence was then amplified by PCR and subcloned into the pET28a(+) vector between the NcoI and SalI sites, placing a 6×His tag at the C-terminus of EGFP. BL21 *E. coli* were transformed with the resulting construct encoding double-tagged Cdc25C (Cdc25C-GFP-His), and recombinant protein expression was induced as described above for GST-tagged proteins.

Bacteria were lysed by sonication in 20 mM Tris-HCl (pH 8.0), 500 mM NaCl, 40 mM imidazole, 1 mM DTT, 1× protease inhibitors, 0.5% Triton X-100, and 1 mg/mL lysozyme. Lysates were clarified by centrifugation at 13,000 × *g* for 15 min at 4°C. Cleared lysates were incubated with 1/20 volume of Ni-NTA agarose (GE Healthcare Life Sciences; 17-5268-01) with rotation for 2 h at 4°C. The beads were washed three times with 20 mM Tris-HCl (pH 8.0), 500 mM NaCl, and 80 mM imidazole, rinsed three times with EB, and eluted with EB supplemented with 300 mM imidazole.

Before fluorescence imaging, purified Cdc25C-GFP-His was diluted 1:10 with EB alone or EB supplemented with 500 mM NaCl or 12.5% PEG-8000, resulting in a final protein concentration of approximately 46 μM. Images were acquired using MetaMorph software (version 7.7.5.0) on a ZEISS Axioplan 2 imaging system equipped with a Plan-NEOFLUAR 40×/1.2 objective.

#### Microinjection and maturation of *Xenopus* oocytes

Capped mRNAs encoding myc- or HA-tagged proteins were generated from pCS2-based expression plasmids using the mMESSAGEmMACHINE® high-yield capped RNA transcription kit. Stage VI *Xenopus* oocytes were manually defolliculated from surgically excised ovaries in modified Barth’s solution, injected with 2-3 ng RNA in 10–20 nL of H_2_O, and matured with 1-3 μM progesterone or left untreated. When ≥80% of progesterone-treated oocytes underwent germinal vesicle breakdown, 6-10 oocytes from each treatment group were collected and homogenized in 60–100 μL EB supplemented with 1 mM DTT, 1 mM ATP, 20 mM NaF, and 0.1 mg/ml each of leupeptin, pepstatin, and chymostatin. Homogenates were centrifuged at ∼14,000 × g for 30 min, and the cytoplasmic layer between the surface lipid and yolk pellet was collected for immunoprecipitation or GST pulldown assays.

#### GST pulldown and immunoprecipitation of *Xenopus* oocyte extracts

For GST pulldown assays, 5 μl of immobilized GST-14-3-3 (0.3-1 μg/μL; an equal-volume mixture of GST-tagged human 14-3-3θ and 14-3-3η) or GST-ΔCycB (0.2 μg/μL; GST-tagged Δ90 sea urchin cyclin B) was incubated with 20 μL of *Xenopus* oocyte extract at 4°C for 4 h. Beads were washed 3-5 times with 500 μL of ice-cold EB containing 300 mM NaCl, 0.1% Triton X-100, and 3 mM DTT. Bound proteins were eluted with SDS-PAGE sample buffer and analyzed by immunoblotting.

Immunoprecipitation of oocyte extracts was performed as described for immunoprecipitation of myc-Cdc25C in Cdc25C activity assays. Antibodies for immunoprecipitation of MAPK were generated by immunizing rabbits with recombinant proteins followed by affinity purification.^20^ Antibodies for immunoprecipitation of CRM1 were purchased from Santa Cruz Biotechnology (sc-74454). Antibodies for immunoprecipitation of RSK1/2 were purchased from Cell Signaling Technology (9355). Anti-HA antibodies used for immunoprecipitation of HA-tagged Plx1 were obtained from Santa Cruz Biotechnology (sc-805).

#### Pulldown of GST-tagged proteins by Cdc25C immunocomplexes

Myc-tagged Cdc25C proteins were immunoprecipitated from TNT products, *Xenopus* oocyte extracts, or lysates of HEK293T cells transiently expressing myc-tagged Cdc25C proteins with anti-Myc antibodies as described in Cdc25C activity assays. Treatment of IP-Cdc25C with IOE and phosphorylation of IOE-Cdc25C with TNT-produced mitotic kinases were also performed as described in Cdc25C activity assays. For pulldown assays, 5 μl of Cdc25C immunocomplexes was incubated with 1 μL soluble GST-Cdc25C_375-550ˆ_ or GST-ΔCycB (0.5 μg/μl) in 40 μL of EB supplemented with 1 mM DTT and protease inhibitor cocktail at 22°C for 2 h. Beads were washed three times with EB, and bound proteins were eluted with 10 μL of 1.5× SDS sample buffer. Eluates were analyzed by immunoblotting with anti-GST or anti-Myc antibodies.

#### SDS-PAGE and immunoblotting

Aliquots (5-10 μl) from each sample set were separated by SDS-PAGE on either 10% gels (30% acrylamide:1% bisacrylamide = 2.57:1) or 12.5% gels (30% acrylamide:1% bisacrylamide = 4:1), alongside prestained molecular weight standards obtained from MilliporeSigma (SDS7B2) or Thermo Scientific (26616). Proteins were transferred to nitrocellulose membranes (Santa Cruz Biotechnology), stained with Ponceau S (Sigma-Aldrich; P7170), and immunoblotted with the indicated primary antibodies.

Primary antibodies were as follows. For Cdk1: Santa Cruz Biotechnology (sc-54). For phospho-Y15-Cdk1: Cell Signaling Technology (9111). For MAPK: Santa Cruz Biotechnology (sc-1647) or polyclonal antibodies generated in our previous study.^20^ For phosphorylated/activated MAPK/ERK2: Cell Signaling Technology (9106). For RSK2: Santa Cruz Biotechnology (sc-9986). For Plx1: Invitrogen (33-1700). For CRM1: Santa Cruz Biotechnology (sc-74454). For phospho-histone H1: Upstate Biotechnology (06-597). For K/HpSP and RXXpS/T motifs: Cell Signaling Technology (9477 and 9614, respectively). For Myc tag: Santa Cruz Biotechnology (sc-40), MilliporeSigma (SAB4301136), or OriGene (TA150121). For HA tag: Santa Cruz Biotechnology (sc-805). For GST tag: Santa Cruz Biotechnology (sc-459) or Huabio (ET1611-47).

Secondary antibodies were HRP-conjugated goat anti-rabbit and goat anti-mouse IgG (Bio-Rad; 1706515 and 1706516), and immunoblots were developed using enhanced chemiluminescence (ECL). For reprobing, membranes were stripped once or twice in buffer containing 2% SDS and 0.7% β-mercaptoethanol in 62.5 mM Tris-HCl (pH 6.8) at 37°C for 45 min. Stripping efficiency was tested by redeveloping blots with ECL.

### Quantification and statistical analysis

Densitometric analysis of immunoblot signals was performed using NIH ImageJ (version 1.44p), and relative values were calculated by normalization to the corresponding controls. For the quantitative analysis presented in Figure S10, data are expressed as mean ± standard deviation (SD) from three independent experiments (*n* = 3). Statistical significance was assessed using Student’s *t*-test, with *p* < 0.01 considered statistically significant.
