# Supplementary material for: Site-comprehensive phosphorylation dismantles a disordered interaction hub to drive Cdc25C activation
Source: iScience. 2026 Nov 5;29(11):118169. doi: 10.1016/j.isci.2026.118169 (PMC13769019; doi:10.1016/j.isci.2026.118169)
Supplement: Supplementary file 1 [file mmc1.pdf]

iScience, Volume *29*

Supplemental information

Site-comprehensive phosphorylation dismantles a disordered interaction hub to drive Cdc25C activation

Tan Tan, Chuanfen Wu, Ruoning Wang, Wei Zhang, Sue-Hwa Lin, and Jian Kuang

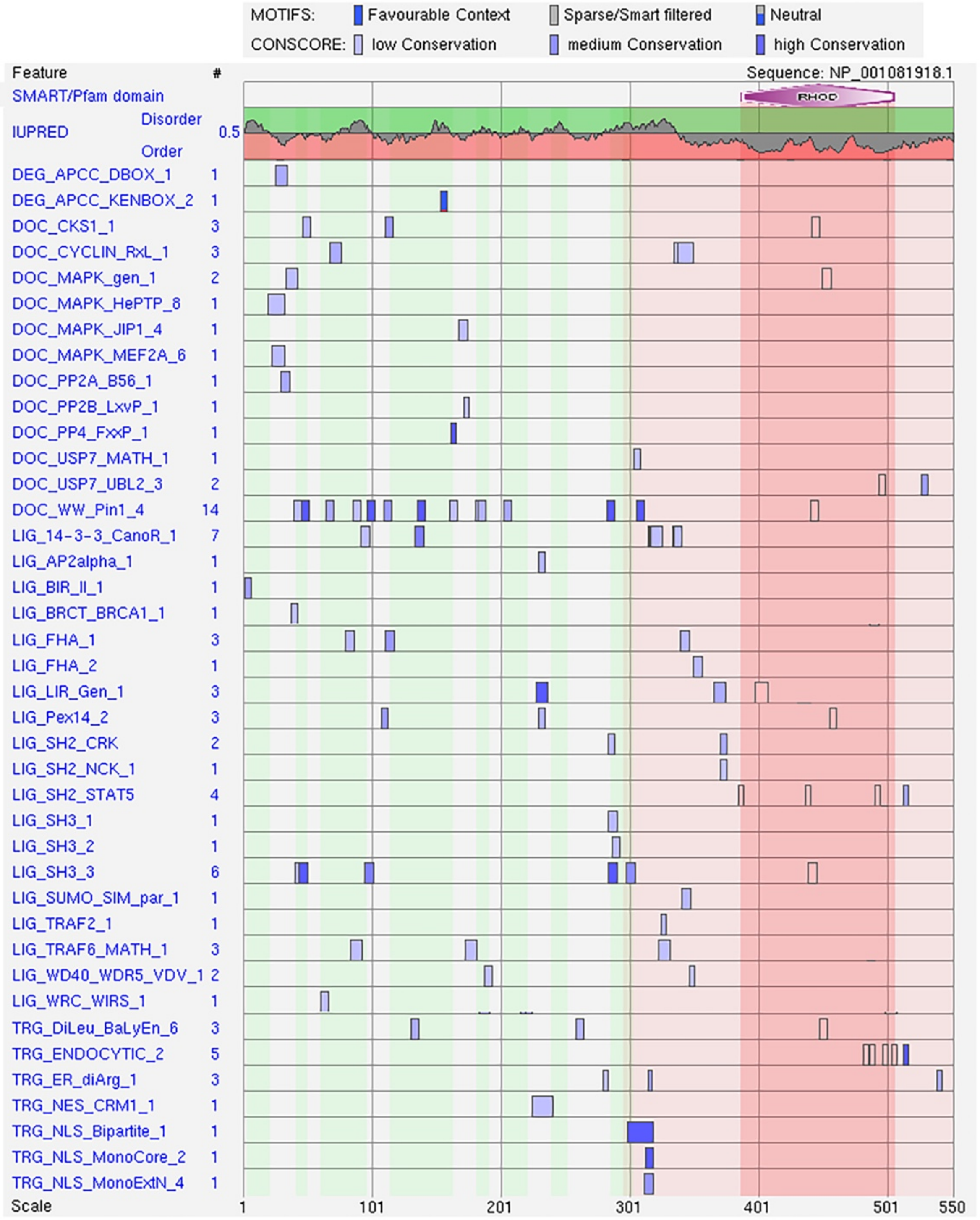

Figure S1. The long IDR of Cdc25C is enriched in short linear motifs, Related to Introduction.
Analysis of the *Xenopus laevis* Cdc25C protein sequence (GenBank: NP_001081918.1) using the Eukaryotic Linear Motif (ELM) resource (http://elm.eu.org) reveals that the long intrinsically disordered regulatory region (IDR) is highly enriched in predicted short linear motifs (SLiMs) that have putative roles in mediating specific protein-protein interactions. The IDR also contains numerous phosphorylation sites (not shown).

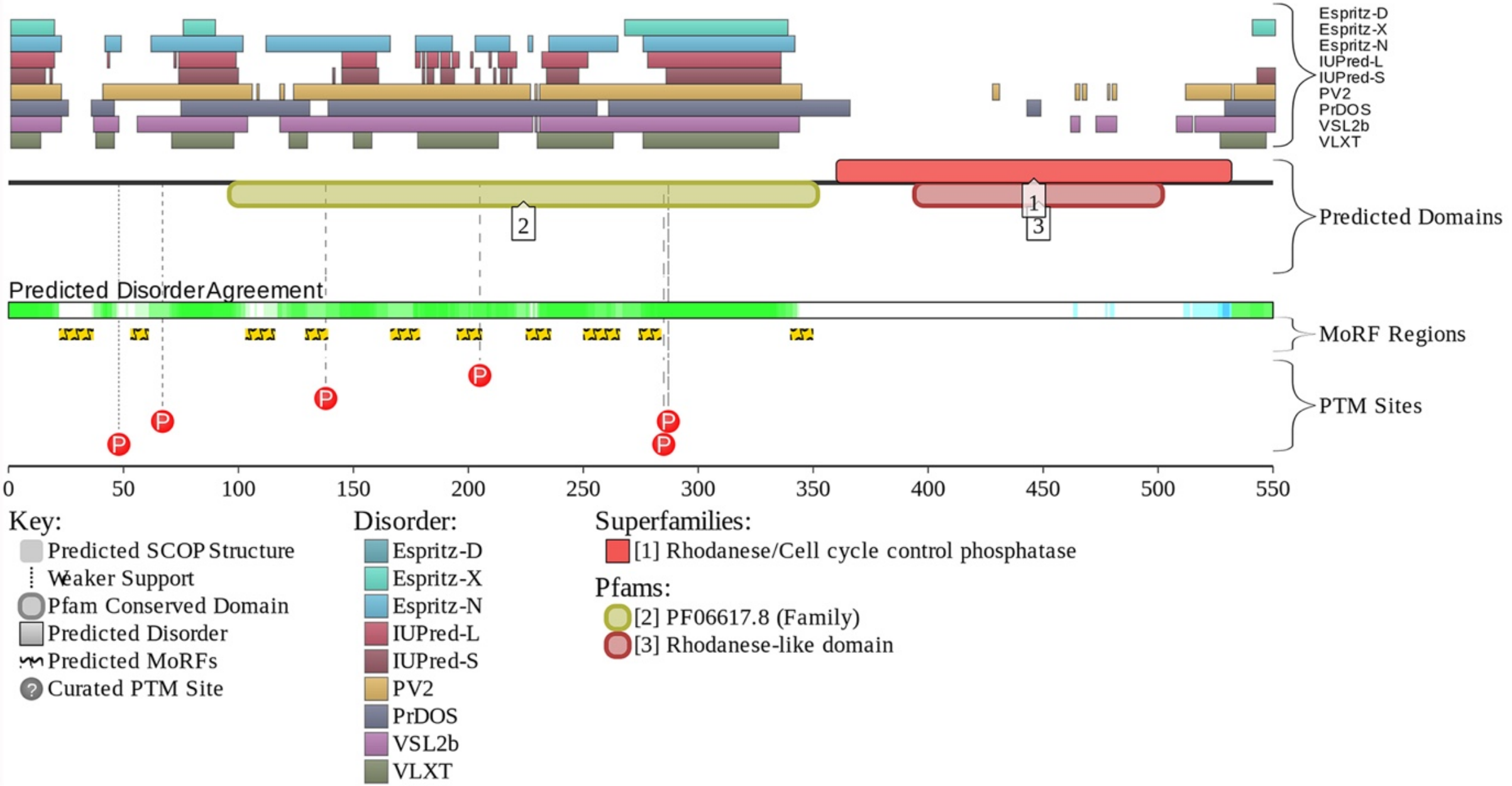

Figure S2. The long IDR of Cdc25C is enriched in molecular recognition feature regions, Related to Introduction.
Analysis of the *Xenopus laevis* Cdc25C protein sequence (GenBank: NP_001081918.1) using the Database of Disordered Protein Predictions (https://d2p2.pro) reveals that the long IDR contains numerous predicted molecular recognition feature (MoRF) regions, which are segments within IDRs, typically spanning 10-70 residues, that undergo disorder-to-order transitions upon binding to partner proteins.

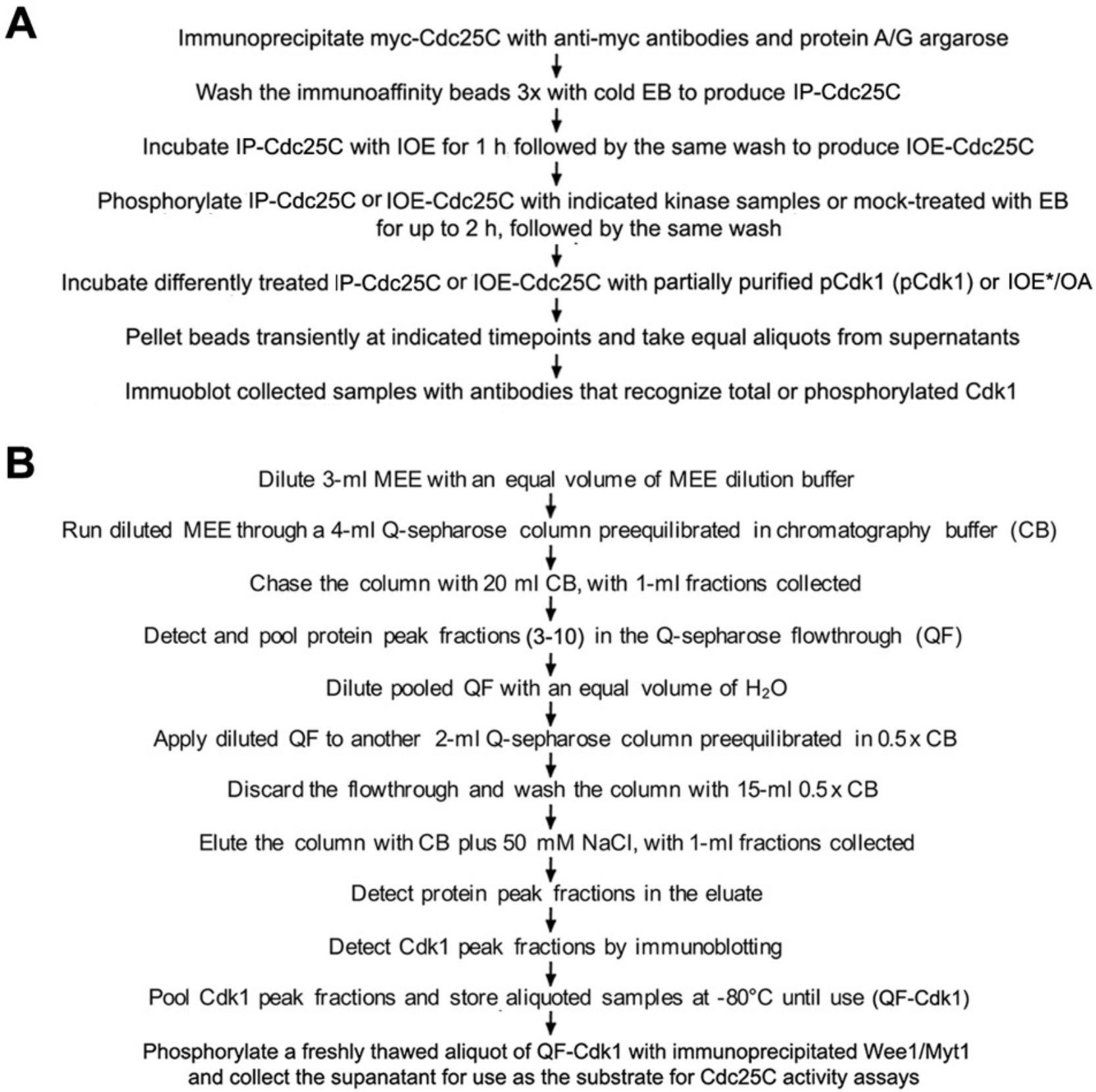

Figure S3. Assays of phosphoregulation of Cdc25C activity in immunocomplexes, Related to Figure 1.
(A) Workflow for assaying the effects of phosphorylation by different kinase samples on Cdc25C activity in immunocomplexes.
(B) Workflow for preparation of partially purified phosphorylated Cdk1 (pCdk1) as the substrate in Cdc25C activity assays.

A

Kinase name	Kinase type	Identified major phosphorylation sites
Cdk1/CycB	Proline-directed kinase	T67, T138, S163, S285, T308
MAPK	Proline-directed kinase	T48, T67, T138, S163, S205
Plx1	Acidophilic kinase	S193, S270, S350 (S38, S61)
RSK2	Basophilic kinase	S317, T318, S319

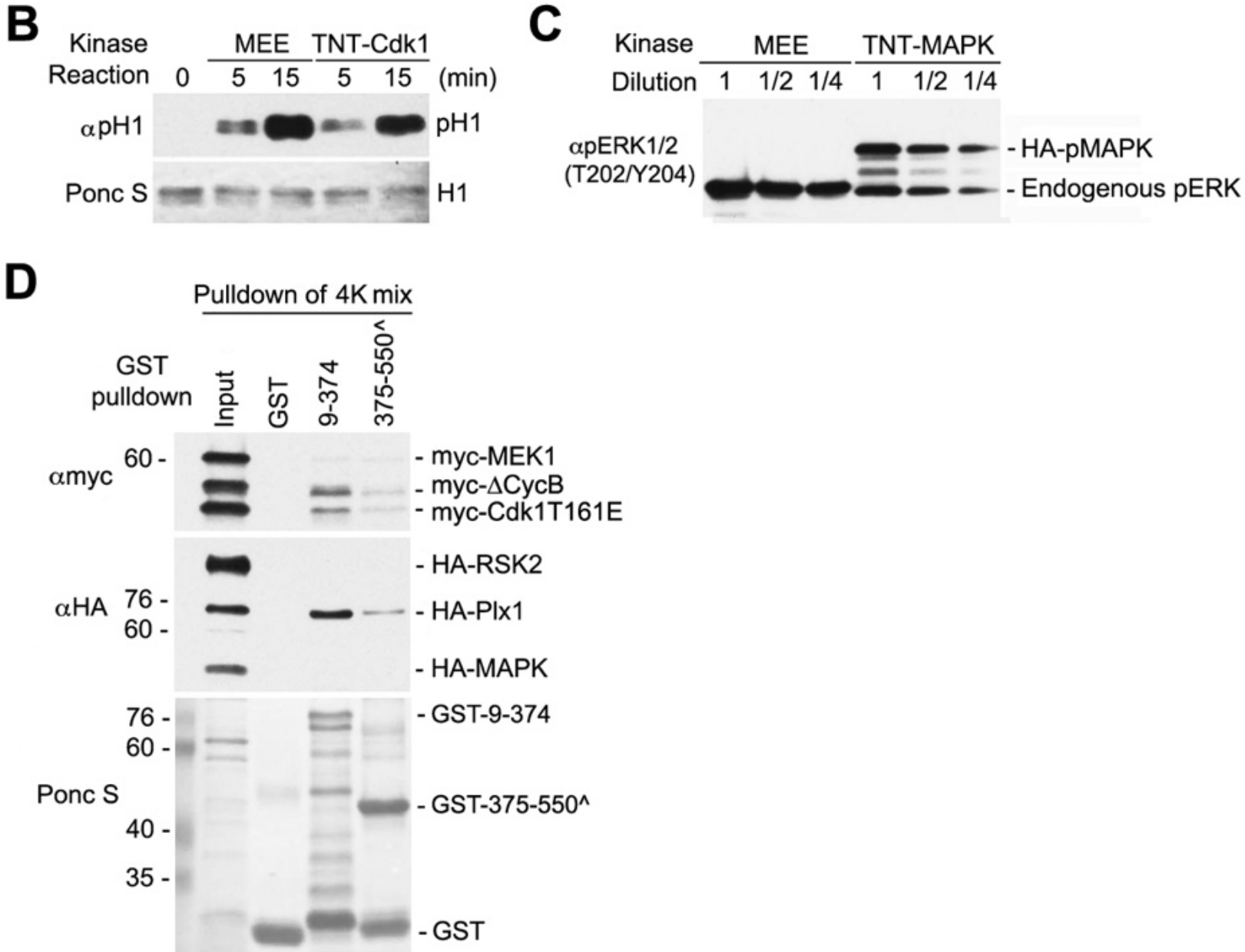

Figure S4. Characterization of TNT-derived mitotic kinases used in this study, Related to Figure 2.

(A) Summary of previously identified phosphorylation sites in Xenopus Cdc25C targeted by the four major mitotic kinases. Additional predicted Plk1 phosphorylation sites within the N-terminal IDR, identified using the ELM resource, are shown in parentheses.

(B) Histone H1 (H1) was phosphorylated with MEE or TNT-produced Cdk1 for the indicated times. H1 phosphorylation was detected by immunoblotting with phosphospecific antibodies, and total H1 was visualized by Ponceau S staining (Ponc S).

(C) Serial dilutions of MEE or TNT-derived active MAPK were analyzed by immunoblotting with phosphospecific antibodies recognizing activated MAPK.

(D) Immobilized GST, GST-Cdc25C$_{9\text{-}374}$ (9-374), or GST-Cdc25C$_{375\text{-}550\wedge}$ (375-550^) was incubated with a TNT-produced mixture of the four major mitotic kinases (4K mix) for 15 min. Input 4K mix (1/20) and bead eluates were analyzed by immunoblotting with the indicated antibodies after Ponc S staining.

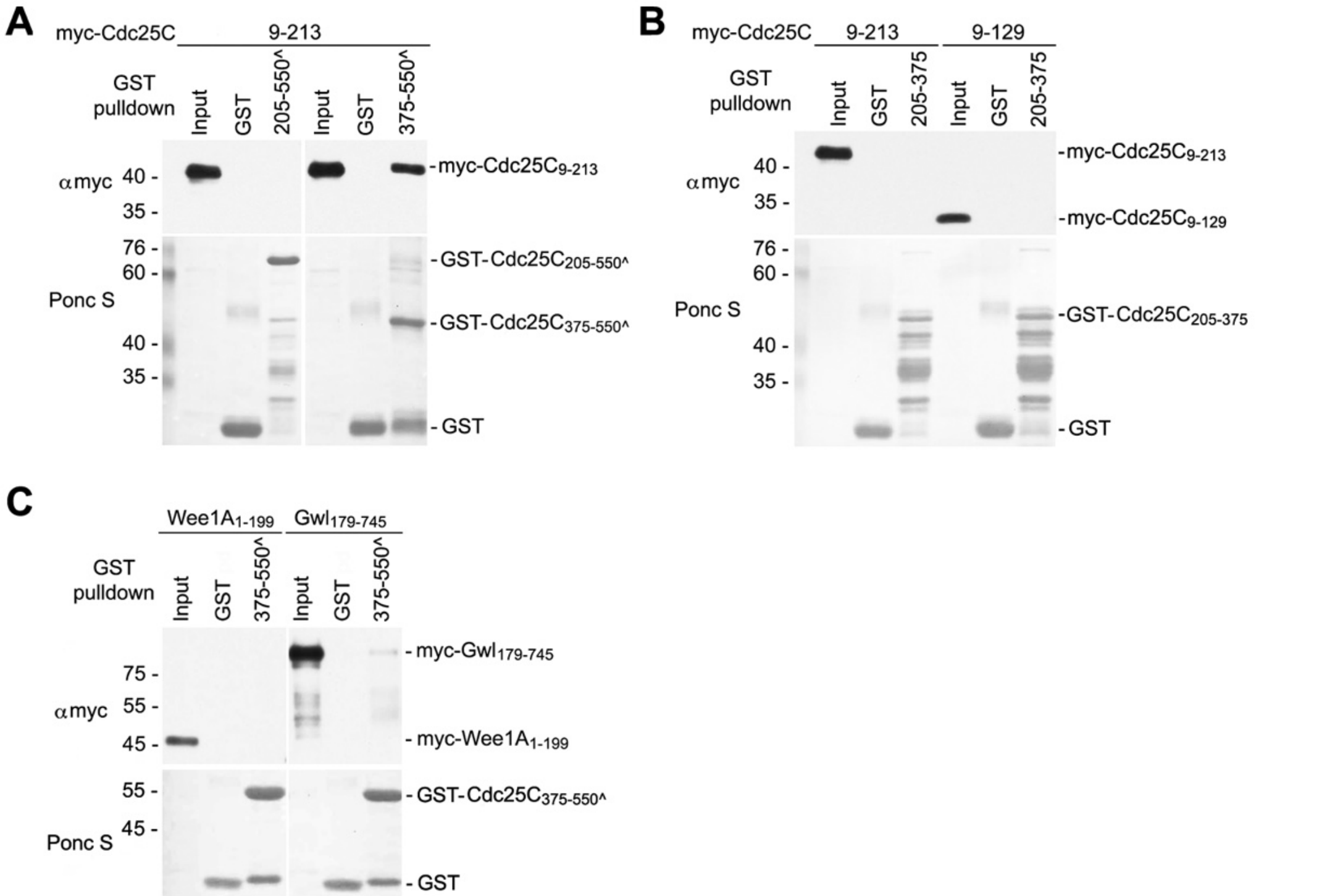

Figure S5. Specificity of the regulatory-catalytic domain interaction of original-state Cdc25C, Related to Figure 3.

(A) Myc-tagged Cdc25C$_{9\text{-}213}$ was incubated either with immobilized GST or GST-Cdc25C$_{205\text{-}550^\wedge}$ or with immobilized GST or GST-Cdc25C$_{375\text{-}550^\wedge}$ for 90 min. Input soluble proteins (1/20) and bead eluates were immunoblotted with anti-Myc antibodies after Ponceau S (Ponc S) staining.

(B) Myc-tagged Cdc25C$_{9\text{-}213}$ and Cdc25C$_{9\text{-}129}$ were incubated with immobilized GST or GST-Cdc25C$_{205\text{-}375}$ for 90 min, and samples were analyzed as in (A).

(C) Myc-tagged Wee1A$_{1\text{-}199}$ and Gwl$_{179\text{-}745}$ were incubated with immobilized GST or GST-Cdc25C$_{375\text{-}550^\wedge}$ for 90 min, and samples were analyzed as in (A).

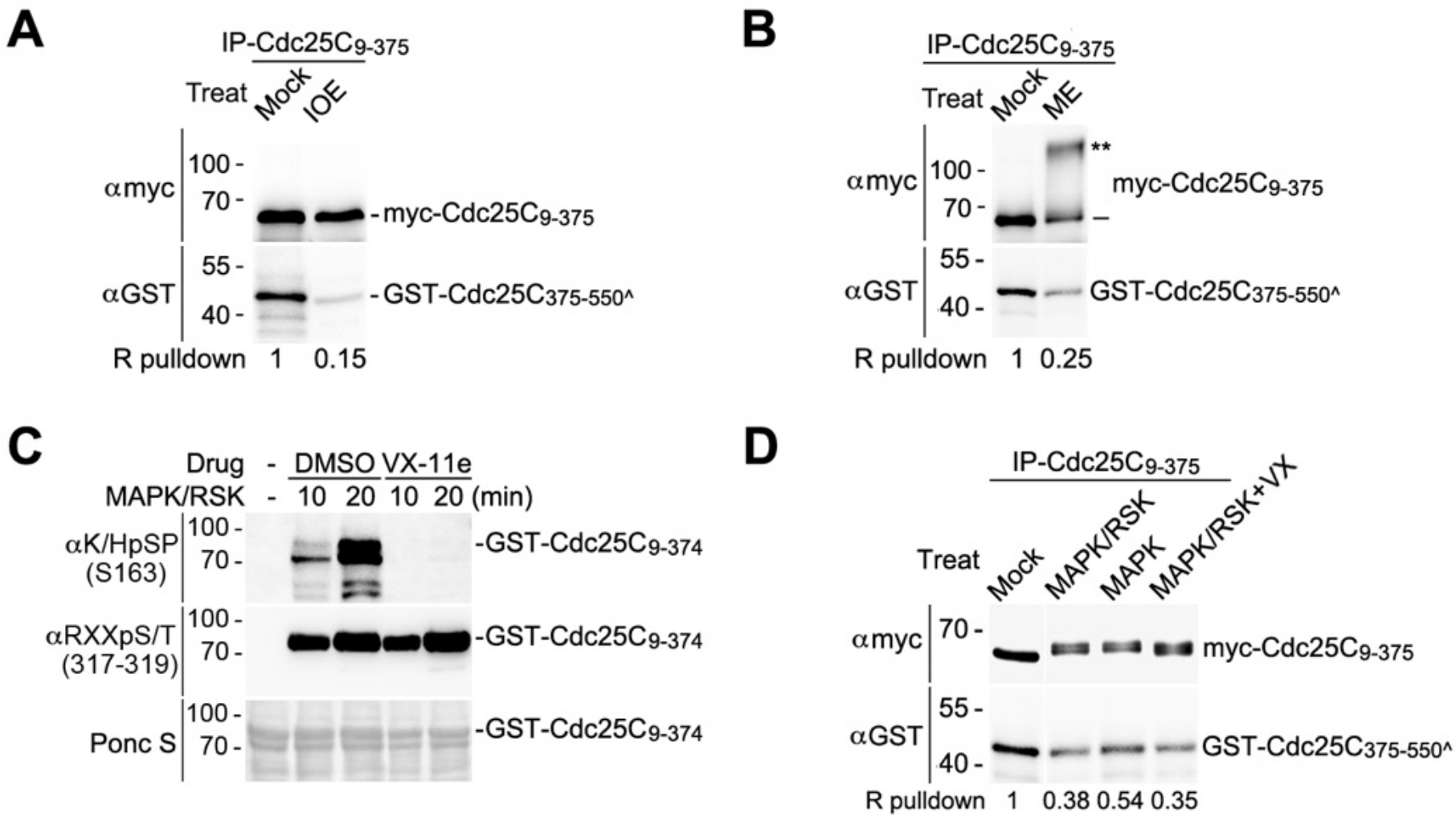

Figure S6. Additional evidence that both G2-state conversion and mitotic phosphorylation weaken the regulatory-catalytic domain interaction of original-state Cdc25C, Related to Figure 3.

(A) Myc-Cdc25C$_{9\text{-}375}$ immunoprecipitated from TNT product (IP-Cdc25C$_{9\text{-}375}$) was treated with IOE for 2 h or mock-treated, followed by incubation with soluble GST-Cdc25C$_{375\text{-}550^\wedge}$ for 1 h. Bead eluates were analyzed by immunoblotting with anti-Myc and anti-GST antibodies. Relative GST-Cdc25C$_{375\text{-}550^\wedge}$ pulldown values (R pulldown) were calculated by normalizing the pulldown signal to that of the mock-treated control.

(B) Myc-Cdc25C$_{9\text{-}375}$ immunoprecipitated from HEK293T cell lysates (IP-Cdc25C$_{9\text{-}375}$) was incubated with mitotic extract (ME) derived from synchronized HEK293T cells for 2 h or mock-treated, followed by incubation with soluble GST-Cdc25C$_{375\text{-}550^\wedge}$ for 1 h. Bead eluates were analyzed as in (A).

(C) Immobilized GST-Cdc25C$_{9\text{-}374}$ was phosphorylated with TNT-derived MAPK/RSK mixture in the presence of the MAPK inhibitor VX-11e or DMSO control for 10 or 20 min. Bead eluates were immunoblotted with the indicated phosphospecific antibodies after Ponceau S (Ponc S) staining.

(D) IP-Cdc25C$_{9\text{-}375}$, prepared as in (B), was mock-treated or phosphorylated with the indicated TNT-derived kinase samples for 2 h and then processed as in (B).

A

```
>NP_001081918.1 M-phase inducer phosphatase 1-B [Xenopus laevis]

                                                                              RxL(1):
 01 MAESHIMSSEAPPKTNTGLNFRTNCRMVLNLLREKDCSVTFSPEQPLTPVTDLAVGFSNLSTFSGETPKR 070
    67-75
 71 CLDLSNLGDETAPLPTESPDRISSGKVESPKAQFVQFDGLFTPDLGWKAKKCPRGNMNSVLPRLLCSTPS 140

141 FKKTSGGQRSVSNKENEGELFKSPNCKPVALLLPQEVVDSQFSPTPENKVDISLDEDCEMNILGSPISAD 210
                                                           MP51 region: 263-315
211 PPCLDGAHDDIKMQNLDGFADFFSVDEEEMENPPGAVGNLSSSMAILLSGPLLNQDIEVSNVNNISLNRS 280
                                                            RxL(2): 334-348
281 RLYRSPSMPEKLDRPMLKRPVRPLDSETPVRVKRRRSTSSSLQPQEENFQPQRRGTSLKKTLSLCDVDIS 350

351 TVLDEDCGHRQLIGDFTKVYALPTVTGRHQDLRYITGETLAALIHGDFSSLVEKIFIIDCRYPYEYDGGH 420

421 IKGALNLHRQEEVTDYFLKQPLTPTMAQKRLIIIFHCEFSSERGPKMCRFLREEDRARNEYPSLYYPELY 490

491 LLKGGYKDFFPEYKELCEPQSYCPMHHQDFREELLKFRTKCKTSVGDRKRREQIARIMKL           550
```

B

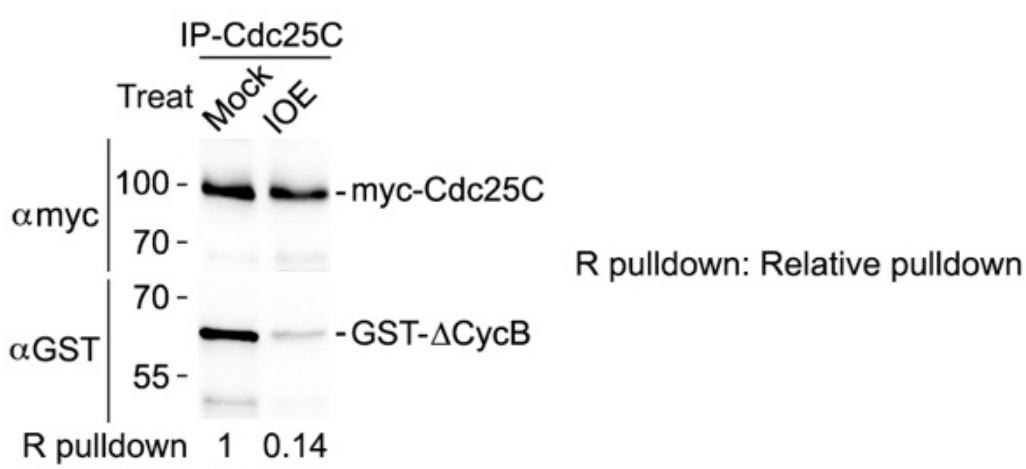

Figure S7. Characterization of Cdc25C interaction with cyclin B, Related to Figure 4.

(A) Potential cyclin B binding regions in Cdc25C. The amino acid sequence of Xenopus Cdc25C is shown, with RxL motifs identified using the ELM resource (see Fig. S1) highlighted in green and the Xenopus Cdc25C sequence corresponding to the human MP51 region previously reported to interact with cyclin B highlighted in yellow.

(B) Myc-Cdc25C immunoprecipitated from HEK293T cell lysates (IP-Cdc25C) was incubated with IOE for 2 h to induce G2-state conversion or mock-treated with EB, followed by incubation with soluble GST-ΔCycB for 1 h. Bead eluates were analyzed by immunoblotting with anti-Myc (myc-Cdc25C) and anti-GST (GST-ΔCycB) antibodies.

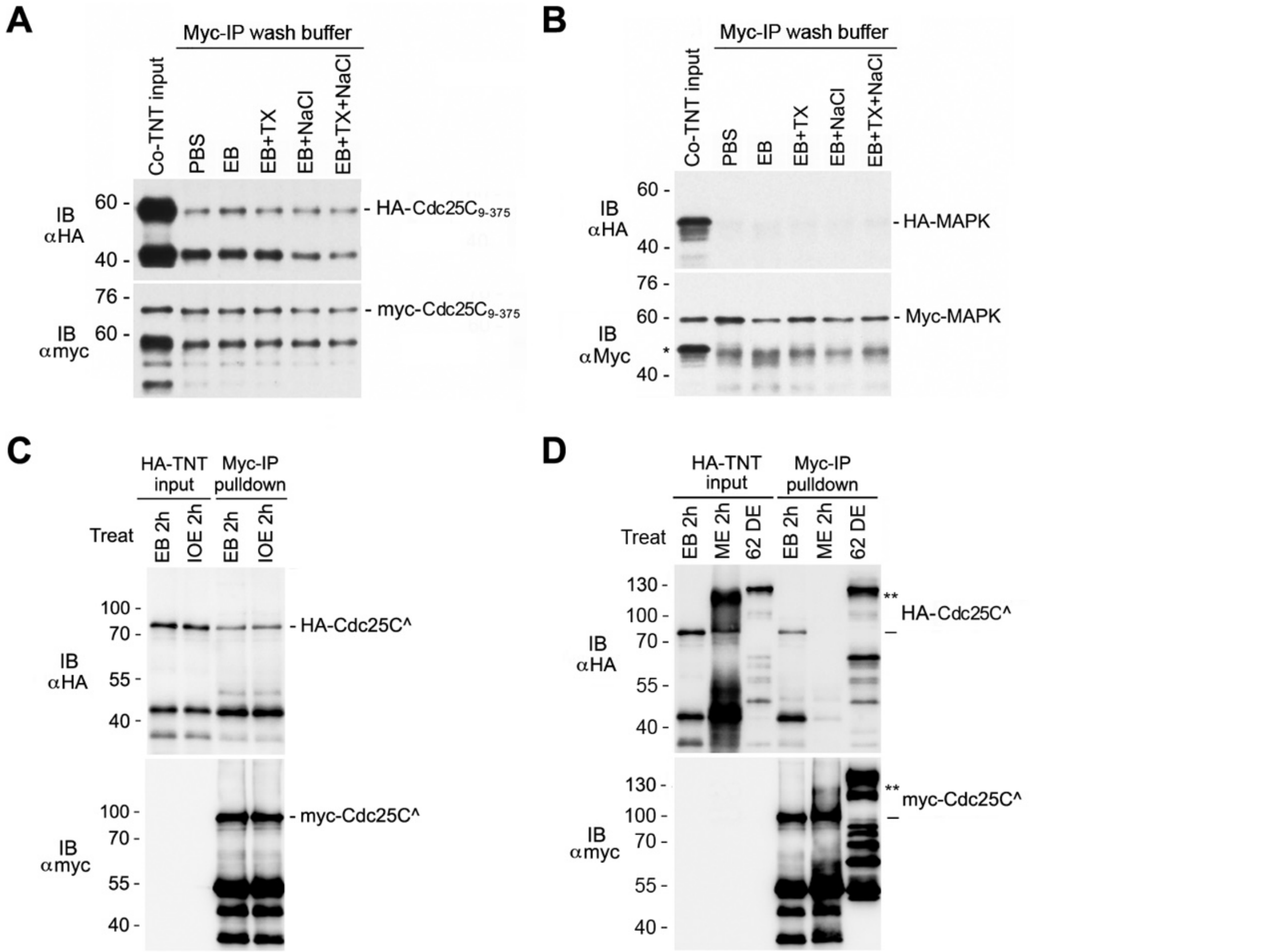

Figure S8. Characterization of self-association of original-state Cdc25C, Related to Figure 5.

(A) Myc-$Cdc25C_{9\text{-}375}$ and HA-$Cdc25C_{9\text{-}375}$ were co-produced by TNT, and the mixture was immunoprecipitated with anti-Myc antibodies. Immunocomplexes were washed twice with the indicated buffers and analyzed by immunoblotting with anti-Myc and anti-HA antibodies. TX, 0.5% Triton X-100; NaCl, 500 mM NaCl.

(B) Myc-MAPK and HA-MAPK were processed as described in (A). The asterisk indicates residual HA-MAPK signals after stripping.

(C) Soluble HA-Cdc25C^ and immunoprecipitated myc-Cdc25C^ were each mock-treated with EB or incubated with IOE for 2 h, and equivalently treated pairs were then incubated together for 1 h. Following two washes of beads with EB, bead eluates (Myc-IP pulldown) were analyzed in parallel with HA-TNT input (1/10) by immunoblotting with anti-Myc and anti-HA antibodies. The results shown are representative of two independent experiments.

(D) Soluble HA-Cdc25C^ and immunoprecipitated myc-Cdc25C^ were each mock-treated with EB or incubated with mitotic extract (ME) derived from synchronized HEK293T cells for 2 h, whereas soluble HA-Cdc25C^62DE and immunoprecipitated myc-Cdc25C^62DE were untreated. Equivalently treated pairs were then incubated together for 1 h, and samples were processed as in (C). The results shown are representative of two independent experiments.

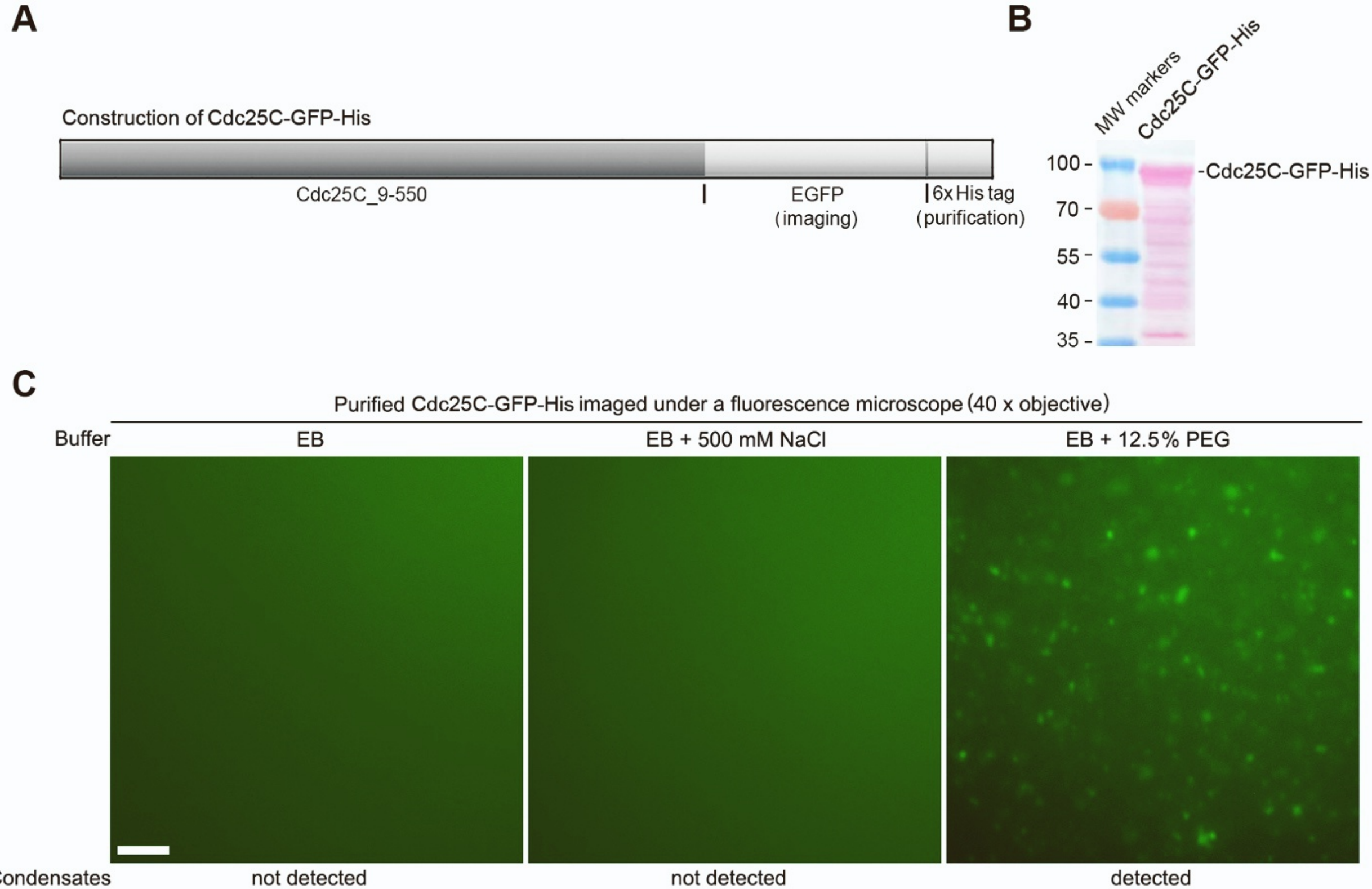

Figure S9. Cdc25C does not form condensates in the absence of macromolecular crowding, Related to Figure 5.
(A) Schematic illustration of recombinant Cdc25C containing C-terminal GFP and His tags (Cdc25C-GFP-His).
(B) Purified Cdc25C-GFP-His was analyzed by SDS-PAGE followed by Ponceau S staining.
(C) Purified Cdc25C-GFP-His (~46 μM) was imaged by fluorescence microscopy in the indicated buffers under identical acquisition settings. No droplet-like condensates were detected in either EB or EB supplemented with 500 mM NaCl. In contrast, droplet-like structures were observed in the presence of 12.5% PEG, consistent with macromolecular crowding-induced condensation. Similar results were obtained with purified Cdc25C$_{9\text{-}375}$-GFP-His. Scale bar, 10 μm (applies to all three images)

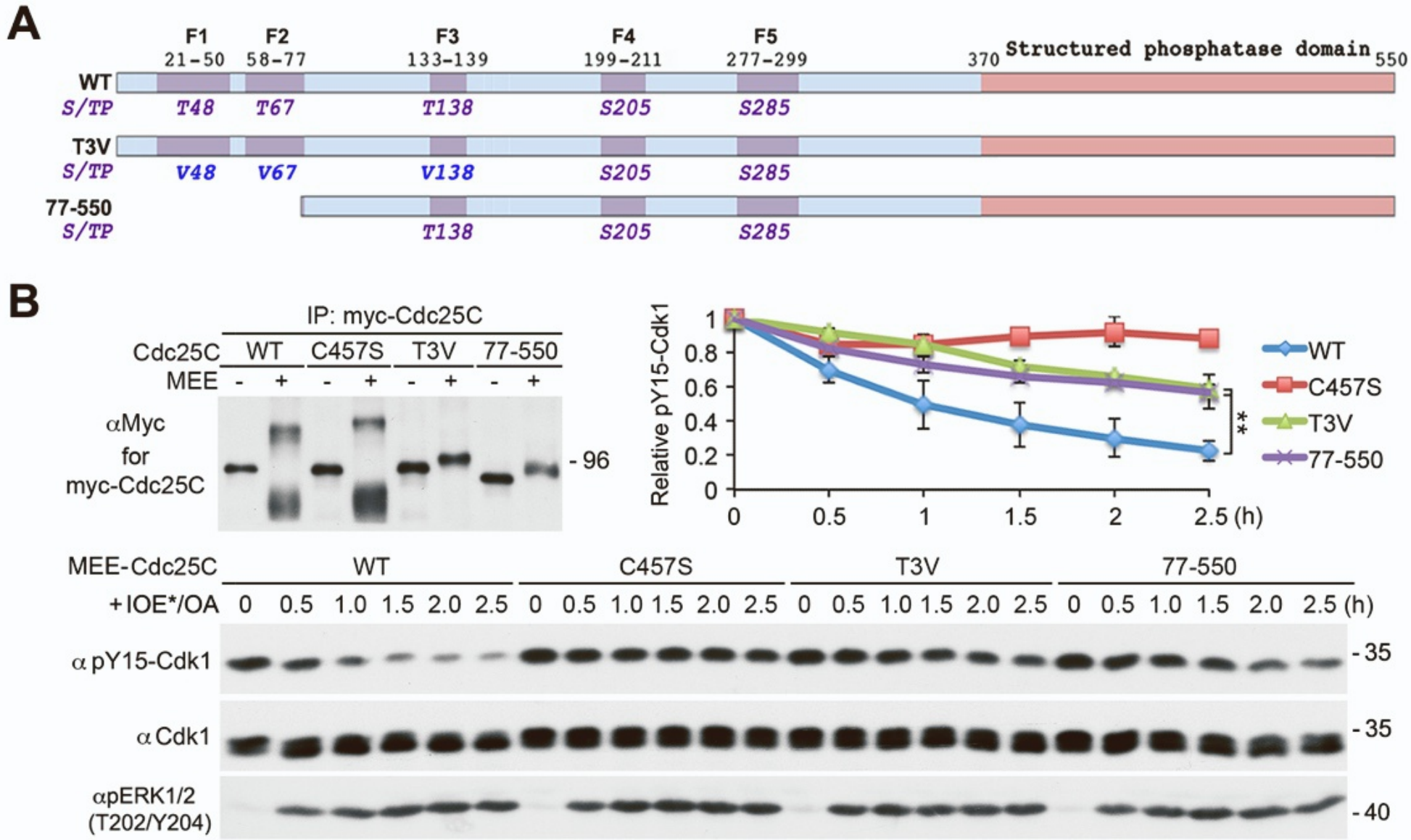

Figure S10. Supershift-defective Cdc25C mutants in phosphoactivation assays, Related to Figure 6.

(A) Schematic of Cdc25C showing five conserved regions containing five conserved S/TP motifs, and the two supershift-defective mutants (T3V and 77-550).

(B) Indicated forms of TNT-produced Cdc25C were first immunoprecipitated and then incubated with MEE for 2 h. Washed immunocomplexes were analyzed for electrophoretic mobility shifts of myc-Cdc25C by anti-Myc immunoblotting (upper left) and for phosphatase activity by measuring Cdk1 Y15 dephosphorylation in 1:10-diluted IOE*/OA for the indicated times (lower panels). The pY15-Cdk1 signals were quantified by densitometry, normalized to total Cdk1, and expressed relative to the corresponding values at 0 h. The upper-right graph shows mean ± SD from three independent experiments (n = 3). Statistical significance was assessed using Student's t-test, comparing T3V and 77–550 mutants individually with WT at 2.5 h (**$p < 0.01$ for each comparison).

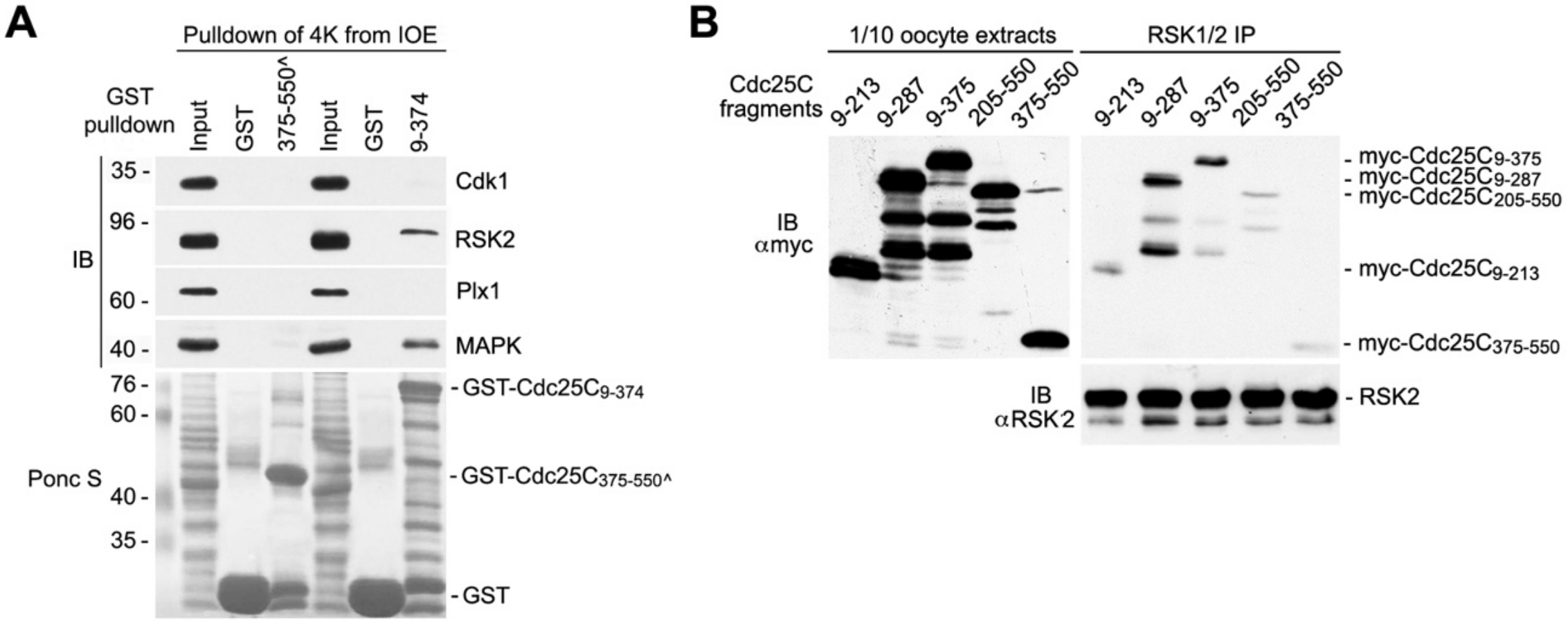

Figure S11. Characterization of interactions between G2-state Cdc25C and the major mitotic kinases, Related to Figure 7.
(A) Immobilized GST, GST-Cdc25C$_{9\text{-}374}$ (9-374), or GST-Cdc25C$_{375\text{-}550\wedge}$ (375-550^) was incubated with IOE for 15 min. Input IOE (1/20) and bead eluates were analyzed by immunoblotting with antibodies for each of the indicated proteins after Ponceau S (Ponc S) staining. The results shown are representative of two independent experiments.
(B) Indicated myc-tagged Cdc25C fragments were ectopically expressed in Xenopus oocytes by mRNA injection. Oocyte extracts were immunoprecipitated with anti-RSK1/2 antibodies. After bead washing, input extracts (1/10) and bead eluates were analyzed by immunoblotting with anti-Myc antibodies, and bead eluates were also probed with anti-RSK2 antibodies.

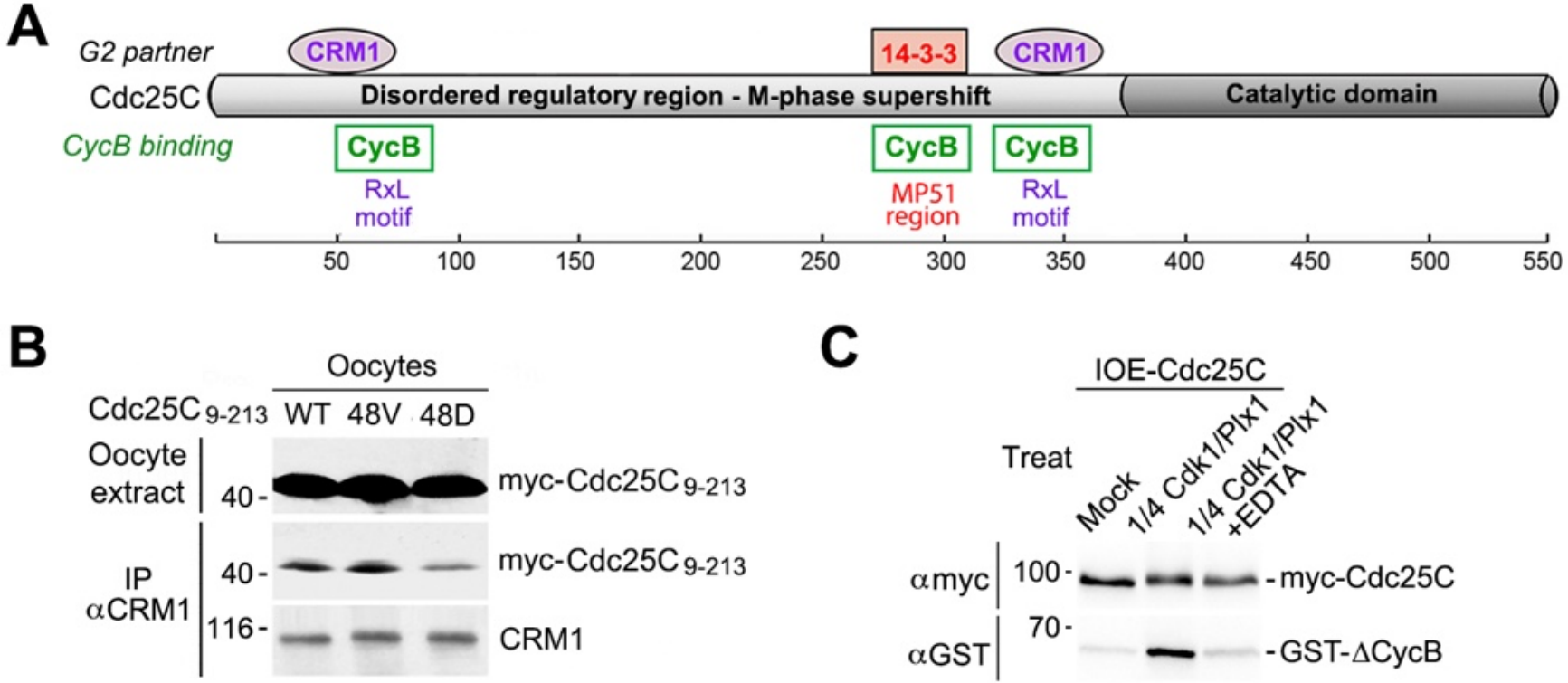

Figure S12. Phosphoregulation of cyclin B interaction with G2-state Cdc25C, Related to Figure 7.

(A) Schematic of docking sites for CRM1 and cyclin B in Cdc25C.

(B) Indicated forms of myc-Cdc25C$_{9\text{-}213}$ were expressed in Xenopus oocytes by mRNA injection, and extracts were immunoprecipitated with anti-CRM1 antibodies. Input extracts (1/10) and bead eluates were analyzed by immunoblotting with anti-Myc antibodies (myc-Cdc25C$_{9\text{-}213}$), and bead eluates were also probed with anti-CRM1 antibodies.

(C) Myc-Cdc25C immunoprecipitated from HEK293T cell lysates was treated with IOE for 2 h to generate G2-state Cdc25C (IOE-Cdc25C) and subsequently incubated with the indicated TNT kinase samples in the presence or absence of EDTA, which inhibits phosphorylation by chelating magnesium. Washed immunocomplexes were then incubated with soluble GST-ΔCycB for 1 h, and bead eluates were analyzed by immunoblotting with anti-Myc (myc-Cdc25C) and anti-GST (GST-ΔCycB) antibodies.

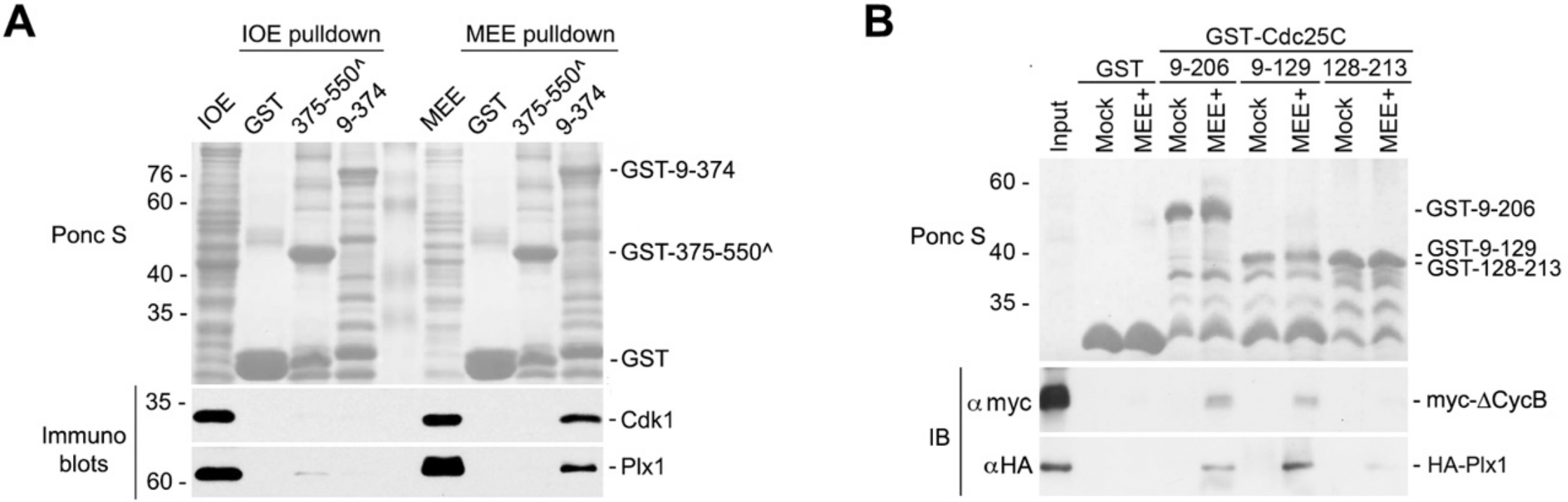

Figure S13. Differential interaction of the Cdc25C IDR with cyclin B and Plx1 in MEE versus IOE, Related to Figure 7.
(A) Immobilized GST, GST-Cdc25C$_{9-374}$ (9-374), and GST-Cdc25C$_{375-550^\wedge}$ (375-550^) were each incubated with IOE (left) or MEE (right) for 15 min. Input IOE/MEE (1/20) and bead eluates were analyzed by immunoblotting with antibodies for each of the indicated proteins after Ponceau S (Ponc S) staining.
(B) Immobilized GST, GST-tagged Cdc25C$_{9-206}$ (9-206), Cdc25C$_{9-129}$ (9-129), and Cdc25C$_{128-213}$ (128-213) were each mock-treated or incubated with MEE supplemented with TNT-produced myc-ΔCycB and HA-Plx1 (MEE+) at 4°C for 30 min. Input MEE+ (1/5) and bead eluates were analyzed by immunoblotting with anti-Myc and anti-HA antibodies after Ponc S staining.

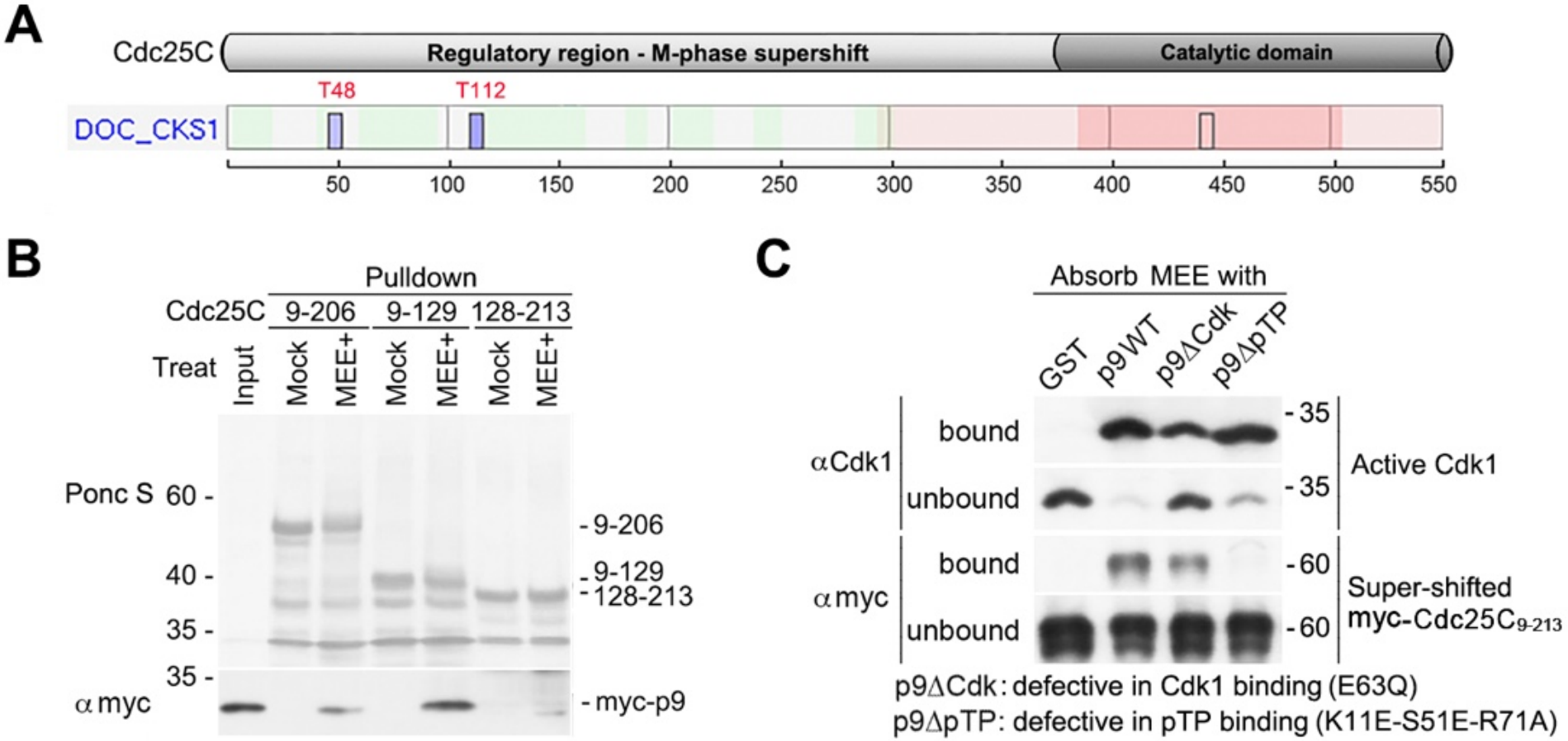

Figure S14. Characterization of p9 interaction with Cdc25C, Related to Figure 7.
(A) Predicted CKS1 binding sites in Xenopus Cdc25C, as shown in Fig. S1.
(B) GST-tagged Cdc25C$_{9\text{-}206}$ (9-206), Cdc25C$_{9\text{-}129}$ (9-129), and Cdc25C$_{128\text{-}213}$ (128-213) were each mock-treated with EB or incubated with MEE supplemented with TNT-produced myc-p9 (MEE+). Input MEE+ (1/5) and bead eluates were analyzed by immunoblotting with anti-Myc antibodies after Ponceau S (Ponc S) staining.
(C) Myc-Cdc25C$_{9\text{-}213}$ was first incubated with MEE for 1 h and then adsorbed with immobilized GST or the indicated forms of GST-p9 at 4°C for 1 h. Following bead washing, both supernatants (unbound) and bead eluates (bound) were analyzed by immunoblotting with anti-Cdk1 and anti-Myc antibodies.

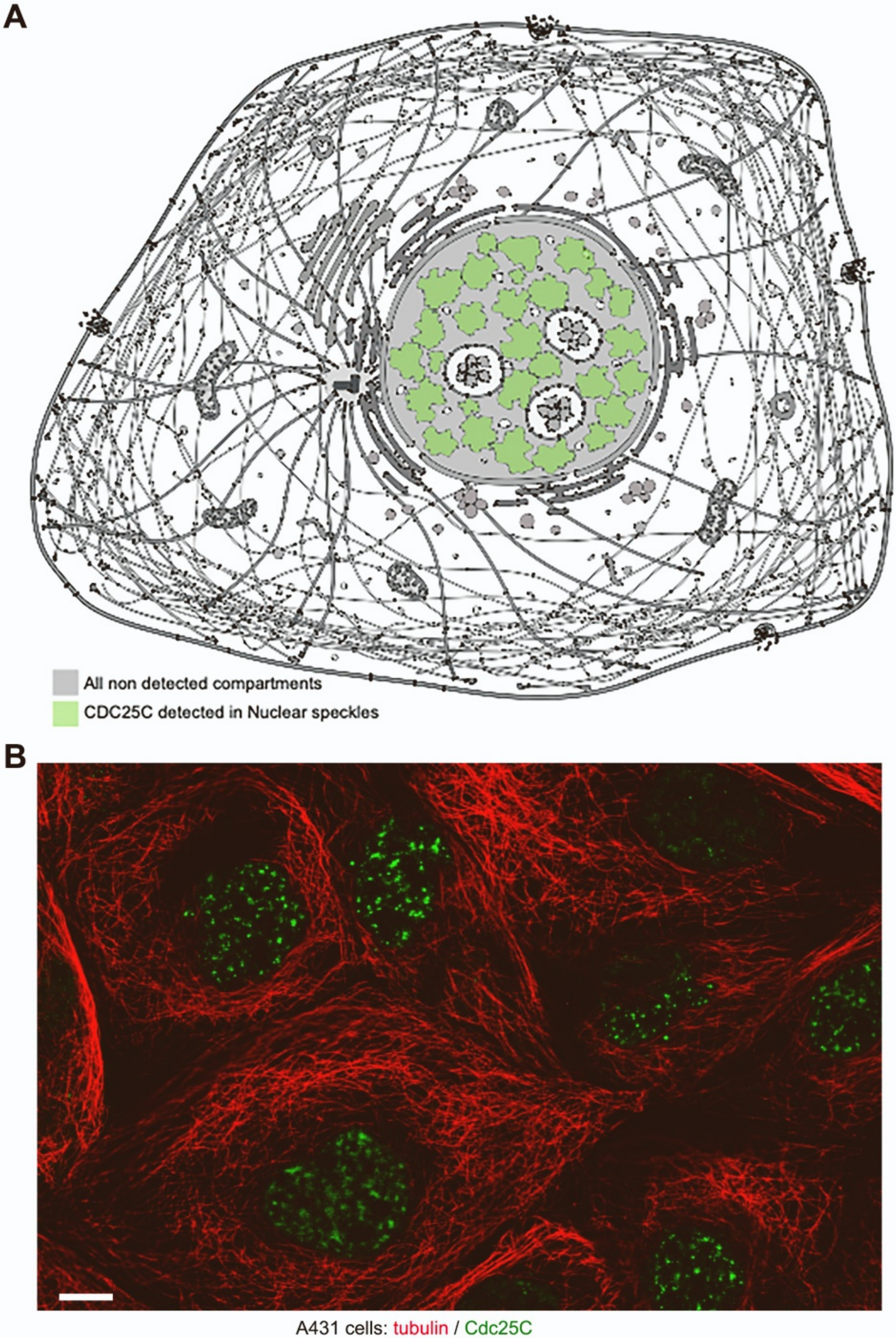

Figure S15. Nuclear speckle localization of human CDC25C, Related to Discussion.

(A) Schematic representation of CDC25C subcellular localization in interphase cells.

(B) Representative immunofluorescence image of A-431 cells stained for CDC25C (green) and tubulin (red). Images and localization data were obtained from the Human Protein Atlas (CDC25C, ENSG00000158402; https://www.proteinatlas.org/ENSG00000158402-CDC25C) [41] and reproduced under the Creative Commons Attribution 4.0 International License. Image credit: Human Protein Atlas. Scale bar, 10 μm.
